# Impact of Sarcopenia on the Severity of the Liver Damage in Patients With Non-alcoholic Fatty Liver Disease

**DOI:** 10.3389/fnut.2021.774030

**Published:** 2022-01-17

**Authors:** Vittoria Zambon Azevedo, Cristina Alina Silaghi, Thomas Maurel, Horatiu Silaghi, Vlad Ratziu, Raluca Pais

**Affiliations:** ^1^Doctoral School Physiology, Physiopathology and Therapeutics 394, Sorbonne Université, Paris, France; ^2^Centre de Recherche de Cordeliers, INSERM UMRS 1138, Paris, France; ^3^Department of Endocrinology, “Iuliu Hatieganu” University of Medicine and Pharmacy Cluj-Napoca, Cluj-Napoca, Romania; ^4^Institute of Cardiometabolism and Nutrition, Paris, France; ^5^Assistance Publique Hôpitaux de Paris, Hôpital Pitié-Salpêtrière, Paris, France; ^6^Department of Surgery V, “Iuliu Hatieganu” University of Medicine and Pharmacy Cluj-Napoca, Cluj-Napoca, Romania; ^7^Sorbonne Université, Paris, France; ^8^Centre de Recherche Saint Antoine, INSERM UMRS 938, Paris, France

**Keywords:** fatty liver, NAFLD, sarcopenia, obesity, muscle-liver axis, myosteatosis, inflammation, sarcopenic obesity

## Abstract

An extensive body of the literature shows a strong interrelationship between the pathogenic pathways of non-alcoholic fatty liver disease (NAFLD) and sarcopenia through the muscle-liver-adipose tissue axis. NAFLD is one of the leading causes of chronic liver diseases (CLD) affecting more than one-quarter of the general population worldwide. The disease severity spectrum ranges from simple steatosis to non-alcoholic steatohepatitis (NASH), cirrhosis, and its complications: end-stage chronic liver disease and hepatocellular carcinoma. Sarcopenia, defined as a progressive loss of the skeletal muscle mass, reduces physical performances, is associated with metabolic dysfunction and, possibly, has a causative role in NAFLD pathogenesis. Muscle mass is a key determinant of the whole-body insulin-mediated glucose metabolism and impacts fatty liver oxidation and energy homeostasis. These mechanisms drive the accumulation of ectopic fat both in the liver (steatosis, fatty liver) and in the muscle (myosteatosis). Myosteatosis rather than the muscle mass *per se*, seems to be closely associated with the severity of the liver injury. Sarcopenic obesity is a recently described entity which associates both sarcopenia and obesity and may trigger worse clinical outcomes including hepatic fibrosis progression and musculoskeletal disabilities. Furthermore, the muscle-liver-adipose tissue axis has a pivotal role in changes of the body composition, resulting in a distinct clinical phenotype that enables the identification of the “sarcopenic NAFLD phenotype.” This review aims to bring some light into the complex relationship between sarcopenia and NAFLD and critically discuss the key mechanisms linking NAFLD to sarcopenia, as well as some of the clinical consequences associated with the coexistence of these two entities: the impact of body composition phenotypes on muscle morphology, the concept of sarcopenic obesity, the relationship between sarcopenia and the severity of the liver damage and finally, the future directions and the existing gaps in the knowledge.

## Introduction

The aging population and the global epidemics of obesity and type 2 diabetes (T2DM) resulted in increased prevalence of several chronic conditions, like non-alcoholic fatty liver disease (NAFLD) and sarcopenia. NAFLD is now recognized as one of the leading cause of chronic liver disease afflicting more than 25.0% of the general population worldwide (1). Sarcopenia, derived from the greek—“*sarcos*” (flesh) and “*penia*” (loss), has been first described in 1989 (2) and is characterized by age-related loss of muscle mass and functional impairment. Since then, numerous studies, definitions and diagnosis criteria have been suggested, leading in 2010 to the development of the first diagnostic consensus of sarcopenia by the European Working Group on Sarcopenia in Older People (EWGSOP) (3). Initially described in older adults (more than 50 millions adults affected in 2010), both sarcopenia and frailty—defined as “decreasing physiologic reserve and increased vulnerability to health stressors” (4)—are now recognized as chronic progressive conditions associated with increased risk of various comorbidities such as chronic liver disease and cardiometabolic disorders, in particular obesity and T2DM, and cancer (5).

Depending on the definitions used, the assessment methods and the study population, sarcopenia is a prevalent condition in patients with cirrhosis (40.0–70.0%) (6, 7) obesity (6.0–43.0%) (8, 9) and NAFLD/non-alcoholic steatohepatitis (NASH) (20.0%) (10). It is likely that NAFLD and sarcopenia share common physiological pathways and are interconnected through the muscle-liver-adipose tissue axis. Skeletal muscle plays a major role in glucose transport and disposal, fatty liver oxidation and energy homeostasis which are all key determinants in the pathophysiology of NAFLD. Ectopic lipid deposition in the skeletal muscle related to increased energy intake causes peripheral insulin resistance (IR) and usually occurs before the onset of NAFLD but is also a common feature of sarcopenia (11). The “metabolic inflexibility” secondary to IR and the crosstalk between the target organs involved (skeletal muscle, liver and adipose tissue) are major determinants in the physiopathology and progression of both conditions (12, 13).

Several studies suggest that sarcopenia is a disease modifier across the spectrum of NAFLD (14). However, the risk of poor clinical outcomes increases when both conditions are associated and therefore is difficult to establish a cause-effect relationship beyond and above the overlap of the common physiopathological pathways. The main topics addressed here are: (i) key mechanisms linking NAFLD and sarcopenia - from IR and low-grade inflammation to myosteatosis and impact of clinical phenotypes on muscle morphology; (ii) clinical evidence linking sarcopenia and NAFLD across the severity spectrum of the liver damage and clinical outcomes; (iii) the concept of sarcopenic obesity (SO); (iv) finally, we will address the future directions and the existing gaps in the knowledge.

## Common Mechanisms Linking Sarcopenia and NAFLD

**Figure 1:**
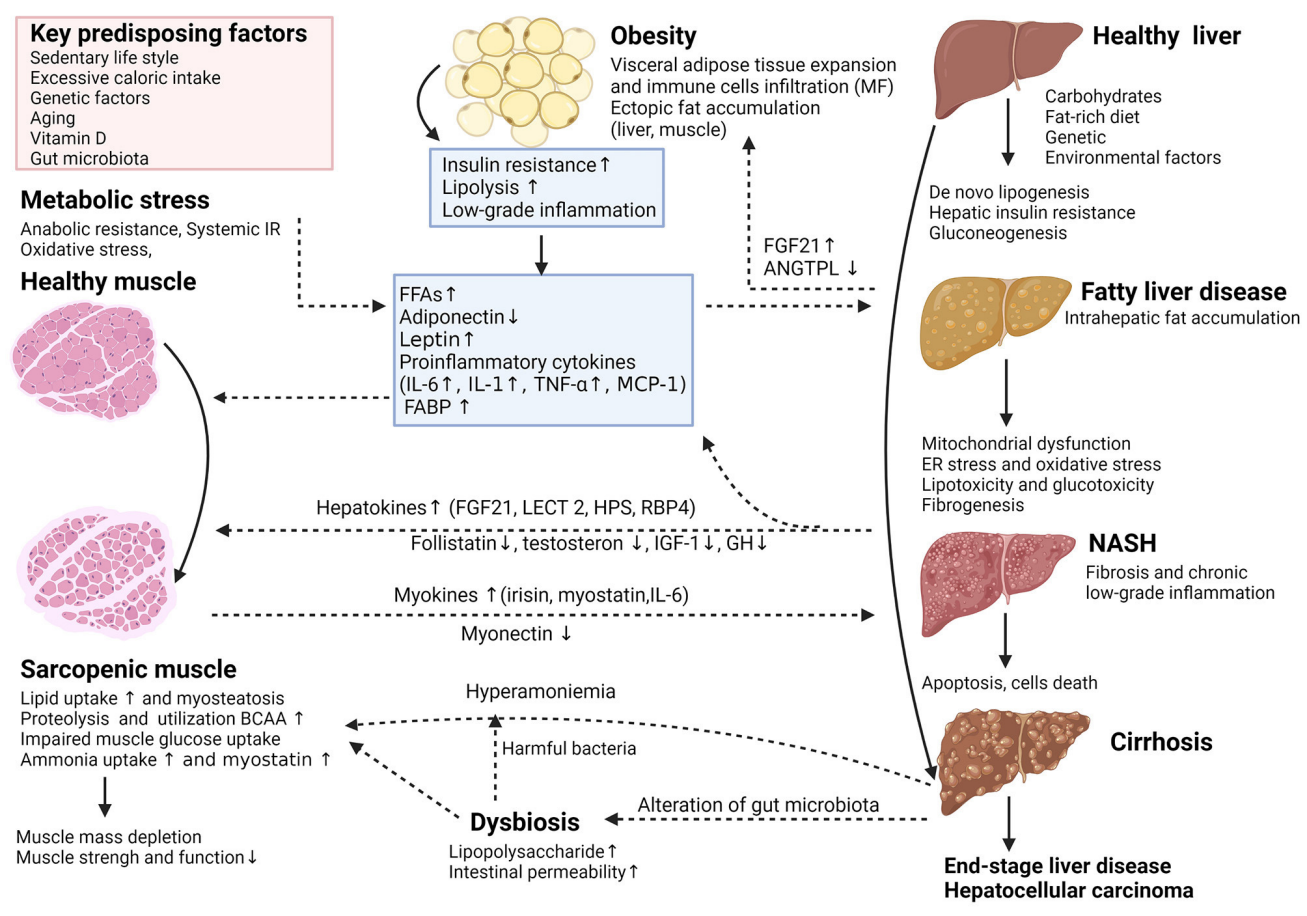
(Created with BioRender.com). Key cellular and molecular mechanisms involved in the complex interplay between adipose tissue, sarcopenia, and the nonalcoholic fatty liver disease (NAFLD). The potential interaction between adipose tissue and skeletal muscle plays an essential role in the pathophysiological and natural course of NAFLD. Adipose tissue dysfunction is characterized by inflammation and adipokine disturbances, subsequent ectopic fat deposition and insulin resistance (IR). In insulin-resistant subjects, insulin fails to promote glycogen synthesis and favors adipose tissue lipolysis, redirecting substrate to “*de novo”* lipogenesis and accelerates proteolysis. These deregulations trigger further insults in hepatocytes through increased inflammation, lipotoxicity, mitochondrial dysfunction, oxidative and endoplasmic reticulum stress and anabolic resistance, which can all contribute to the progression of NAFLD. The release of multidirectional molecular signals comprising myokines and hepatokines regulates a range of systemic metabolic processes including skeletal muscle and hepatic IR, escalating dysfunction of the adipose-muscle-liver axis. Other mechanisms like dysbiosis related to changes in gut microbiota may have additional detrimental effects. Thus, dysregulation of the complex physiological relationship between skeletal muscle and the liver is reciprocally unfavorable and supports each other in a vicious circle, potentially playing a causative role in NAFLD incidence or progression. ANGPTL4, angiopoietin-like 4; BCAA, branched-chain amino acids; ER, endoplasmic reticulum; FABP, fatty acid-binding protein; FFA, free fatty acid; FGF21, fibroblast growth factor 21; GH, growth hormone; HPS, hepassocin; IGF-1, insulin-like growth factor 1; IL-6, interleukin-6; LECT2, leukocyte cell-derived chemotaxin-2; MCP-1, monocyte chemoattractant protein 1; MF, macrophages; NAFLD, nonalcoholic fatty liver disease; NASH, nonalcoholic steatohepatitis; RBP4, retinolbinding protein 4; TNF-α, tumor necrosis factor-alpha.

Extensive studies carried out in recent years showed a strong interrelationship between the pathogenic pathways of non-alcoholic fatty liver disease (NAFLD) and sarcopenia through the muscle-liver-adipose tissue axis. The potential interactions between the adipose tissue and the skeletal muscle may play an essential role in the physiopathology and the natural history of NAFLD. The interplay between muscle and liver is influenced by several factors; among them, insulin resistance (IR), obesity, chronic low-grade inflammation, and several hepatokines and myokines have a significant impact on both entities. Other key factors that may significantly impact the muscle-liver crosstalk are vitamin D deficiency, unhealthy/diet composition, oxidative stress, aging, physical inactivity, and several hormonal changes [growth hormone (GH), insulin-like growth factor 1 (IGF-1), testosterone and osteocalcin] (5, 15). Based on the existing data, the association between sarcopenia and NAFLD seems to be independent of IR or obesity (10, 16, 17). Figure 1 summarizes the key cellular and molecular mechanisms involved in the complex interplay between adipose tissue, sarcopenia, and NAFLD. The dysregulation of the physiological relationship between the skeletal muscle and the liver is bidirectional and potentially plays a role in the progression of NAFLD. However, whether NAFLD directly contributes to sarcopenia or vice versa is still of debate. Further studies are required to specifically focus on the possible mechanisms linking sarcopenia and NAFLD (18).

### Insulin Resistance

#### At the Skeletal Muscle Level

Given that sarcopenia is a multifactorial condition (8, 19), characterized by generalized and progressive loss of skeletal muscle mass (SMM), reduced physical performance and strength (3), it may be exacerbated by the association with some comorbidities that have common pathophysiological backgrounds. Moreover, a condition known as sarcopenic obesity (SO) proves that the detrimental effects of sarcopenia are magnified when associated with obesity. As such, IR is considered not only the core common pathological mechanism responsible for developing sarcopenia, but also a pivotal mechanism in the development and progression of NAFLD/NASH (20, 21).

In addition, the skeletal muscle is the major participant in the whole-body insulin-mediated glucose homeostasis and has the highest requirement of postprandial glucose in an insulin-dependent manner. In the settings of normal skeletal muscle physiology, insulin stimulates glycogen synthesis in the liver and muscle. It also binds to the transmembrane insulin receptor and activates the Protein Kinase C (PKC) pathway, supporting the translocation of the glucose membrane transporter-4 (GLUT4) and thus facilitating glycogen synthesis. Moreover, insulin plays a major role in protein metabolism, promotes the transport of amino acids (AA) in tissues, improves protein synthesis and the inhibition of proteolysis (22, 23), mainly via the p38 mitogen-activated protein kinase (MAPK) and the mammalian target of rapamycin p70S6 kinase (mTOR/p70S6) pathways (24–26).

Skeletal muscle is significantly involved in the energy metabolism. In a healthy subject, fasting periods are characterized by a decrease in insulin levels and by the use of fatty acids (FA) as the preferred fuel substrate (27). After a meal, insulin levels rise, favoring cellular glucose uptake and shifting the fuel source from FA to glucose. *Metabolic flexibility* represents this capacity of recognizing the appropriate fuel substrate and periodically shifts between FA (during fasting) and glucose (postprandial) in order to produce energy (28, 29). The absence of appropriate periodic shifts in glucose and fat oxidation in accordance with the energy state results in *metabolic inflexibility* (30). In these settings, insulin pulsatile secretion is impaired and leads to hyperinsulinemia and insulin resistance (30, 31). IR can develop before NAFLD, as a result of excess adiposity, or may occur subsequent to NAFLD, due to hepatic lipid infiltration leading to impaired glucose and triglycerides metabolism (32). Myosteatosis is a consequence of excessive circulating FA and IR. Muscle cell lipid infiltration is associated with reduced protein synthesis (33, 34).

The skeletal muscle protein synthesis depends on the proper stimulation by anabolic hormones (insulin, GH, IGF-1, sex hormones), AA availability and muscle contractibility (mechanical stimulation) (35, 36). Protein catabolism develops in the context of energy, AA or hormonal deficiencies, physical inactivity, and inflammatory processes (35, 37). The main causes of anabolic resistance include older age, obesity, IR reduced AA availability and systemic inflammation. Very important, in hepatic disorders (NAFLD, NASH, cirrhosis), the decline in the muscle mass is often caused by IR. This leads to anabolic resistance, the loss of protein synthesis in the skeletal muscle (particularly structural muscle proteins, including myosin) and reduced insulin-mediated suppression of protein catabolism which exacerbate proteolysis and lipolysis (38–40). As IR promotes a decrease in SMM, it also worsens sarcopenia and further favors the increased fat accumulation (myosteatosis) (41, 42). As such, the diminished additional SMM reduces insulin-mediated glucose disposal and contributes to IR, promoting gluconeogenesis which, in turn, may exacerbate proteolysis and muscle depletion, resulting in a vicious cycle (43–45). Several studies evaluated the regulatory action in protein synthesis of leucine and arginine. Leucine is involved in protein turnover through protein synthesis stimulation, proteolysis inhibition, and activation of the mammalian target of rapamycin complex 1 (mTORC1) (46, 47). Arginine is an AA used as a substrate for nitric oxide synthesis, which increases the muscle blood flow and further promotes glucose uptake, fatty acid oxidation and lipolysis (48). Several interventional studies showed that AA supplementation may ameliorate sarcopenia by improving the lean body mass (LBM) and the Liver Frailty Index (5, 49). Moreover, anabolic resistance may also be alleviated with exercise (50).

Skeletal muscle lipid levels (or intramyocellular lipids/IMCL) develop particularly when the inflow of free fatty acids (FFAs) exceeds the oxidative capacity of the skeletal muscle. It is now accepted that IMCL accumulation is negatively correlated with insulin sensitivity, suggesting the involvement of IMCL in the development of lipid-induced IR, probably due to a disbalance in the skeletal myocellular lipid influx, lipid metabolism, and oxidative capacity (51). Based on available evidence, the accumulation of bioactive DAG species, at specific intracellular sites, rather than the total levels of IMCL, could have had a role in lipid-induced IR by activating the serine phosphorylation of insulin receptor substrates-1 (IRS-1), and interfering with phosphoinositide 3-kinase (PI3K) activation (24, 52). The accumulation of lipid intermediates causes lipotoxicity by promoting reactive oxygen species (ROS) production and endoplasmic reticulum (ER) stress, which, in turn, lead to impaired mitochondrial dysfunction (53) and create a vicious cycle of lipotoxicity (54). More recent data suggest that intramyocellular lipid dynamics [decreased triglyceride (TAG) synthesis or increased TAG lipolysis, and reductions in oxidative capacity] may represent essential causes in regulating lipid-induced IR (51). Moreover, intermyocellular adipose tissue (IMAT) and IMCL secrete myostatin, C-C motif chemokine ligand 2 (CCL2), tumor necrosis factor-alpha (TNF-α), interleukin-1 alpha (IL-1α), and interleukin-6 (IL-6), thus inducing IR and lipotoxicity (55).

#### At the Hepatic Level

IR plays an important role in NAFLD pathogenesis. IR triggers adipokine-induced liver damage through increased inflammation, oxidative and ER stress, mitochondrial dysfunction, anabolic resistance, and increased deposition of ectopic fat. All of the above mentioned mechanisms are responsible for the progression of NAFLD (56, 57) and account for the overlap between the muscle and the liver damage.

The hepatic fat content is the strongest predictor of IR both in the skeletal muscle and in adipose tissue. In addition, hepatic steatosis and IR are correlated independently of adiposity, which indicates that liver fat, rather than body fat in general, is responsible for this association (58–61). Consequently, DAGs levels are strong predictors of hepatic IR (62, 63) and trigger IR through the activation of the PKC pathway.

The presence of hepatic steatosis is an independent predictor for incident T2DM (64–66). As already shown (67), adipose tissue IR was correlated with the severity of muscle and liver insulin sensitivity, as well as with hepatic steatosis. Moreover, their presence worsens with the progression of glucose intolerance, which may aggravate lipotoxicity, explaining the higher difference between adipose tissue IR, presented by individuals with T2DM and with or without NAFLD. As higher muscle mass is related to greater insulin sensitivity and lower risk of prediabetes, sarcopenia may be an early predictor of diabetes susceptibility, independently of obesity (68). Thus, sarcopenic patients had higher amounts of total body fat mass (FM), more components of metabolic syndrome (MetS), HOMA-IR index, and higher C-reactive protein (CPR) levels, compared to those without sarcopenia (10). Furthermore, a negative relationship was found between HOMA-IR, liver attenuation index and skeletal muscle mass index (SMI), in opposition to a positive correlation between HOMA-IR and CPR, suggesting a potential inflammatory role of the muscle-liver axis.

Consequently, the current therapeutic focus is to reduce the low-grade inflammation through lifestyle changes and physical activity, by promoting visceral fat and fatty liver reduction in association with greater energy expenditure and increased SMM (69). Given the limited expandability of the adipose tissue, excessive lipids accumulation occurs in other compartments such as the liver and skeletal muscle. Thus, during aerobic exercise, the stored lipids are used as a fuel source (70), which can lead to decreased levels of myostatin (15). Myostatin has been suggested as a diagnostic biomarker to predict obesity-associated comorbidities, due to its increased concentration in skeletal muscle and its significant correlation with IR severity (71).

### The Role of Adipose Tissue in Sarcopenia and NAFLD

Adipose tissue may interfere with the liver and muscle through the secretion of various adipokines, such as leptin and adiponectin. Hyperleptinemia is positively correlated with fat mass (FM) (72) and may facilitate IR, liver inflammation and fibrosis (73). It has been shown that circulating leptin levels were higher in patients with NAFLD than in normal subjects, and were positively correlated with the severity of the liver damage (74). Increased leptin levels are found in sarcopenic patients despite their low body FM. The appendicular skeletal muscle mass (ASM) was independently and negatively correlated with leptin even after adjusting for body FM (75).

Adiponectin, another major adipokine, is a protein exclusively secreted from the adipose tissue and negatively correlates with fat accumulation. Adiponectin promotes insulin sensitivity by facilitating glucose uptake in the skeletal muscle and adipose tissue and increases fatty acid oxidation (76, 77). Accordingly, it is likely that adiponectin triggers the preferential use of FFA as a fuel in the skeletal muscle (76–78). Additionally, adiponectin exerts an anti-inflammatory effect (79) and has a hepatoprotective role in liver inflammation and cell injury (80–82).

To sum up, the complex interplay between the adipose tissue, the skeletal muscle and the liver is leading to the development of NAFLD and its progression to NASH, but also to the loss of the skeletal muscle mass and worsening sarcopenia.

### Chronic Low-Grade Inflammation

Both NAFLD and obesity are now recognized as subclinical inflammatory conditions (83, 84). Obesity enlarged adipose tissue secretes adipokines and proinflammatory cytokines which facilitate infiltration of macrophages (MF) and other inflammatory cells. MF are the major source of inflammatory mediators and the importance of macrophage-mediated inflammation is recognized as a cause of IR (84, 85). Thus, MF change the phenotype to M1 and liberate pro-inflammatory factors such as TNF-α, interleukin-1 beta (IL-1β), IL-6, and CCL2 (5, 24, 86, 87) resulting in toxic effects on myocytes and ultimately sarcopenia. It seems that these cytokines induce muscle atrophy by favoring apoptosis and upregulating proteasomal decay of filament proteins (5, 24, 76). IL-6 and TNF-α are able to inhibit the anabolic hormone IGF-1 activity and to induce IR, leading to a catabolic state and reduced myogenesis (76, 88). TNF-α might also induce apoptosis in muscle cells (89). In addition, IL-6 may be secreted by the skeletal muscle during the physical exercise and after its outflow in the blood stream increases liver gluconeogenesis and adipose tissue lipolysis (76, 90). These data were supported by a study showing that CRP and IL-6 were positively correlated with body mass index (BMI) and FM and were inversely correlated with fat-adjusted ASM (91). However, no significant associations were found between CRP and IL-6 levels and obesity or sarcopenia showing that the role of inflammatory cytokines in the development of SO is poorly understood. Similarly, NAFLD is accompanied by hepatic inflammation and IR (92). MF and Kupffer cells contribute to the overall inflammatory environment of the liver and are thought to contribute to decreased hepatic insulin sensitivity by secreting pro-inflammatory molecules that activate pathways involved in insulin signaling. Inflammatory cytokines favor “*de novo*” lipogenesis (92, 93) and increased intra hepatocyte levels of ceramide which may decrease insulin signaling (92, 94) by inhibiting the activation of AKT/PKB (protein kinase B).

Thus, the development of chronic inflammation and oxidative stress induced by the multidirectional molecular signals of cytokines secreted in an excessive manner (80, 81) could aggravate the dysregulation of the muscle-liver axis, resulting in loss of the muscle mass (14) but also playing a causative role in NAFLD progression (5).

### Other Key Factors

Nonetheless, muscle-liver crosstalk is influenced by numerous other factors, such as vitamin D deficiency, hormonal changes (GH; IGF-1; testosterone levels), low physical activity, aging, and diet composition (5, 15).

#### Hepatokines

Hepatokines are liver-secreted proteins that signal through autocrine, paracrine and endocrine signaling to impact hepatic and non-hepatic metabolic processes. The hepatocyte protein secretome undergoes marked changes in response to liver steatosis (95). The most studied hepatokines are: hepassocin (HPS), adropin, angiopoietin-like protein 4 (ANGPTL4), sex hormone-binding globulin (SHBG), fetuin-A and -B, retinolbinding protein 4 (RBP4), selenoprotein P, fibroblast growth factor 21 (FGF21), leukocyte cell-derived chemotaxin 2 (LECT2). HPS or hepatocyte-derived fibrinogen-related protein can mediate IR in both liver and skeletal muscle and increases hepatic lipid accumulation and NAFLD activity scores (96). Furthermore, increased HPS levels in hepatocytes induce IR in the skeletal muscle through the epidermal growth factor receptor/ c-Jun N-terminal kinases (JNK) pathway (97). Increased Fetuin-A in steatosis stimulates pro-inflammatory cytokine production from adipocytes and MF but also may cause IR (98, 99). Administration of FGF21 in mice improves hepatic and peripheral insulin sensitivity (100), suppresses lipolysis in adipose tissue (101), and reduces triglyceride and DAG levels in liver and skeletal muscle (102, 103). However, these beneficial effects on obesity, T2DM, and fatty liver disease seem to contrast with the high levels of FGF21 identified in these disorders, being currently difficult to specify whether this reflects a resistance to FGF21 or it is a compensatory reaction to basal metabolic stress (95).

LECT2 positively correlates with obesity and severity of liver steatosis and mediates skeletal muscle IR and hepatic IR (103–105). RBP4 is secreted by hepatocytes and adipose tissue and is increased in steatosis. RBP overexpression is related to inflammation and IR in mice and humans and has been validated as a biomarker for a series of metabolic diseases, including T2DM and obesity (106, 107). Selenoproteins P induces peripheral and hepatic IR and is considered a biomarker for a range of disorders including NAFLD, obesity, T2DM, and cardiovascular disease (CVD) (108). Withal, the liver-specific deletion of the gene encoding selenoprotein P enhances insulin signaling in muscle and liver and improves whole-body glucose tolerance (109). Positive metabolic actions are registered for adropin, a hepatokine that improves insulin sensitivity, hepatic steatosis and reduces adiposity. Low levels of adropin are linked to whole-body adiposity, hepatic steatosis, IR, and CVD (110–112). ANGPTL4 is generally produced in hepatic and adipose tissue and is decreased in NAFLD. ANGPTL stimulates adipose tissue lipolysis, increases plasma levels of lipids and may cause liver steatosis (113, 114).

Altogether, hepatokines impact hepatic and non-hepatic metabolic disorders and are important drivers of metabolic processes, especially of the IR, directly influencing the pathophysiology of the different components of the muscle-liver axis.

#### Myokines

Myostatin is a myokine known to play a crucial role in the negative regulation of muscle mass. Increased levels of myostatin promote protein catabolism, inhibit growth of skeletal muscle and associate with obesity and IR (15). It has been shown that deletion of myostatin in mice increases muscle mass and reduces adiposity, increases insulin sensitivity and glucose uptake and protects from hepatic steatosis (115–117). Myostatin has both local and endocrine effects that can link sarcopenia and NAFLD via a complex process involving several cellular signaling pathways, resulting in the downregulation of the expression of myogenic factors, the decrease in protein synthesis, and the activation of proteasome–ubiquitin ligases (5, 115). Follistatin is a specific inhibitor that binds myostatin and inhibits its activity by preventing its attachment to the receptor. It has been recently shown that myostatin may be a key molecular mediator of muscle-liver crosstalk. Myostatin modulates the biologic properties of human stellate cell (HSC) in a profibrogenic fashion via activation of JNK and might be a novel muscle-to liver pathway implicated in the pathogenesis of hepatic fibrosis in NAFLD (118).

Other myokines that might link sarcopenia and NAFLD are irisin and myonectin. Irisin improves glucose metabolism, increases adipocyte energy expenditure, modulates the expression of enzymes that inhibit lipid accumulation and reduces weight and has a positive effect on hepatic steatosis (24, 119, 120).

Myonectin promotes fatty acid uptake and links skeletal muscle to lipid homeostasis in the liver and the adipose tissue in response to alterations in the energy state, revealing a novel myonectin-mediated metabolic circuit (121).

#### Vitamin D

Recent studies suggest that vitamin D deficiency or its impaired signaling are involved in metabolic disorders, related to both muscle and the liver (5, 122). It has been shown that vitamin D nuclear receptors (VDRs) are present in human skeletal muscle. Also, the vitamin D signaling by VDRs is involved in myogenesis, myoblast proliferation, and differentiation and in the skeletal muscle growth. Vitamin D levels are considerably lower in individuals with sarcopenia, independently of the presence of obesity (17, 123). Supplementation in vitamin D increases the expression of VDRs in skeletal muscle and improves sarcopenia (124), reinforcing the link between the two entities. Large meta-analyses suggested that daily vitamin D supplementation was beneficial for muscle strength, gait and balance (125) decreasing the risk of falling, especially in those with a baseline vitamin D level of <25 nmol/L (126). On the other hand, recent studies (127) suggest that vitamin D deficiency is independently associated with the severity of the injury in NAFLD. Altogether, these data strengthen the hypothesis that vitamin D is a key mediator in the nexus of NAFLD and sarcopenia and may be a potential promising therapeutic target.

#### Physical Activity and Unhealthy Diet (Lifestyle Changes)

Reduced physical activity correlates independently with sarcopenia in patients with NAFLD (128). The physical exercises required to improve sarcopenia in NAFLD patients should aim to enhance muscle strength, muscle mass, and physical performance. A meta-analysis evaluating the effects of the physical exercise on sarcopenia in patients with NAFLD revealed that endurance and combined (endurance and resistance) exercises improved physical function but have no effect on the muscle mass (129). Endurance exercises increase oxygen consumption, mitochondrial synthesis and skeletal muscle capillaries, improving the cardiovascular system and energy levels (130, 131). Still, the best type of training that improves all three parameters of sarcopenia needs to be defined.

An unbalanced diet, rich in lipids, fructose or sucrose, plays an important role in the occurrence of NAFLD, by favoring the development of subcutaneous and visceral obesity, hypertension, IR, dyslipidemia and hyperuricemia (132, 133). Interestingly, a week of high-fructose diet (>1.5 g/kg/day) can double the intrahepatocellular lipids. During the same time lapse, a fat-rich diet has a similar effect, leading to a 90.0% increase of intrahepatic fat, while a high glucose intake (3.0 g/kg/day) causes a 60.0% increase (134). Nevertheless, a reduction of fructose intake by 50.0% improves the hepatic fat content, plasma transaminases, BMI, and the glucose metabolism (135). The nutritional management in the presence of SO involves FM reduction combined with an increase in muscle mass and strength. These cases should benefit from protein supplementation in order to prevent the catabolism and the muscle loss (136). Patients with sarcopenia and NAFLD cirrhosis require a protein intake of 1.5–2.0 g/kg/day (137). Moreover, in cases of SO, a fairly hypocaloric diet and a late evening snack (50.0 g carbohydrate ± 20.0 g protein) to minimize overnight fasting and prevent muscle destruction, are advised (138, 139).

#### Hormonal Imbalance

It is now widely accepted that older age is correlated to the development of NAFLD in the general population (43). Aging is also associated with a decrease in anabolic hormones (GH, IGF-1, testosterone), which further impacts muscle loss (140). Aging and ectopic fat deposition cause a decline in GH levels, which consequently downregulates the PI3K-AKT/PKB-mTOR pathway, leading to lower levels of IGF-1 and impaired protein synthesis in the muscles (141–143). Plasma IGF-1 levels are positively correlated with LBM and muscle activity and negatively associated with FM. IGF-1 binding proteins (IGFBP), which are produced by the liver, also affect IGF-1 biological activity (144). Moreover, reduced GH and IGF-1 levels favor ectopic fat storage in the liver, contributing to NAFLD development (142, 145).

Under normal circumstances, testosterone increases muscle mass by promoting protein synthesis, skeletal muscle cell expression of androgen receptors, and IGF-1 secretion (36). However, through an increased aromatase activity, obesity favors testosterone aromatization to estradiol, causing hypogonadism (146). Hypogonadism favors central obesity by increasing TNF-α and IL-6, thus contributing to SO (140, 147). Similarly, in menopausal women, the low levels of estrogen and high levels of follicle-stimulating hormone (FSH) and androgens promote SO (148, 149).

## Clinical Evidence Linking NAFLD and Sarcopenia

The largest amount of data linking sarcopenia and NAFLD comes from cross-sectional cohort studies, most of them performed in Asian populations. Several large meta-analyses showed that the risk of NAFLD, NASH and significant fibrosis was increased 1.5–2.5 fold among individuals with sarcopenia (150–153). Conversely, subjects with NAFLD have significantly lower SMI when compared to controls (153). Although these studies outline the bidirectional relationship between sarcopenia and NAFLD, the causal relationship is difficult to establish. The main limitations of these studies come from the significant heterogeneity in the study population and in the definitions and methods used to diagnose both NAFLD and sarcopenia. These shortcomings explain at least in part the differences in the prevalence of sarcopenia across studies (Supplementary Table 1). Most of the studies defined sarcopenia by low-muscle mass, determined by various methods—dual energy-ray absorptiometry (DXA), bioelectric impedance analysis (BIA), CT, and MRI. Despite an international consensus to include muscle strength when available (3), only a minority of studies assessed muscle function and composition.

**Figure 2:**
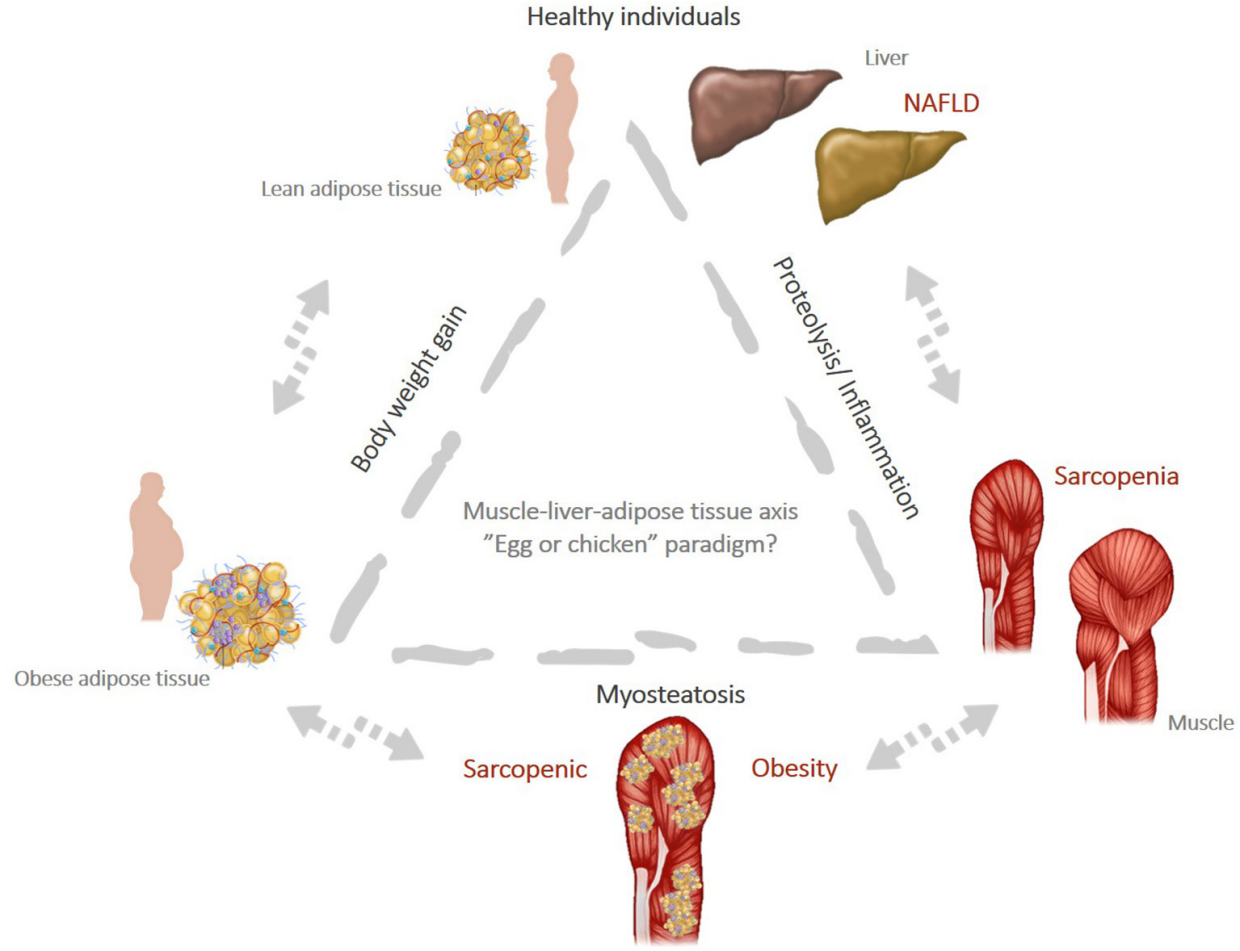
(Created with PowerPoint Microsoft). Muscle-liver-adipose tissue axis: the “egg or chicken” paradigm: weight gain leads to obesity and favors the onset of chronic inflammation that can trigger increased muscle wasting (proteolysis) and promote “*de novo*” lipogenesis pathways. This increase the risk of NAFLD and sarcopenia and favor the milieu for myosteatosis, a condition strictly associated with SO.

Muscle composition, in particular muscle fat infiltration, also called myosteatosis, is a major determinant not only for muscle strength and function, but also for metabolic and liver-related clinical outcomes (144, 154, 155). Thus, adverse muscle composition (AMC) has been widely studied, in an attempt to elucidate the sarcopenia paradigm in NAFLD (144, 156, 157), as shown in Figure 2. Patients with NAFLD have more frequent AMC when compared with patients without NAFLD. A recent study has shown that the skeletal muscle fat index (SMFI) as a reflection of absolute high muscle fat content, rather than a low muscle mass, is strongly and independently associated with the severity spectrum of NAFLD and progressively increases from patients without fatty liver to patients with isolated steatosis, NASH without fibrosis and NASH with significant fibrosis respectively, *p* <0.0015 (155). Another study has shown that NAFLD patients with low muscle mass and high intramuscular fat represent a distinct clinical phenotype with significantly worse metabolic outcomes, particularly with respect to T2DM and coronary heart disease (156). Conversely, patients with NAFLD and normal muscle composition had similar metabolic outcomes as patients with normal liver and normal muscle composition. Very important, this study showed that only a proportion of patients with NAFLD and AMC had sarcopenia according to the classical definition which strengthens the evidence that this particular “unhealthy” phenotype is clearly underestimated by the current methods used to diagnose and assess sarcopenia (156). Altogether, these data suggest that muscle fatty infiltration might be a potential marker associated with the severity spectrum of NAFLD. Remarkably, a recent experimental study showed that myosteatosis is strongly correlated with the severity of the liver damage and discriminates between simple steatosis, NASH and normal liver (144). Thus, myosteatosis has been suggested as a new non-invasive biomarker of NAFLD/NASH, able not only to predict the severity of the liver damage but also to identify early changes muscle composition (144, 157, 158).

The differences in the study population with respect to ethnicity and gender directly impact the body composition and thus limits the generalizability of the results. Most of the studies have been performed on Asian populations (10, 14, 17) and only a minority have been performed in Europe (159) or in the US (160–162). One study performed in the US has shown that the prevalence of sarcopenia was higher in females, in Hispanics and non-Hispanics whites (>40.0%) compared to non-Hispanic Black and non-Hispanic Asian (<10.0%) (161). Several Asian studies derived from the Korean National Health and Nutrition Examination Survey (KHANES) 2008–2011 showed that patients with sarcopenia have a two- to five-fold increased risk of NAFLD (10, 45) and two-fold increase risk of significant fibrosis (45) independently of obesity, IR, MetS and liver enzymes. However, most of these studies used non-invasive methods to diagnose NAFLD and to assess its severity (either biological biomarkers or imaging methods) (14, 155, 159, 160, 163) (Supplementary Table 1) which may lead to the misclassification of NASH and advanced fibrosis.

More recently, the association between sarcopenia and the severity spectrum of NAFLD has been confirmed in a cohort of 309 patients with available liver histology. The prevalence of sarcopenia almost doubled along the severity spectrum of NAFLD and ranged from 9.0% in controls to 18.0% in patients with isolated fatty liver, and 35.0% in patients with NASH. Among individual histological features of NASH, sarcopenia was associated with the amount of steatosis and ballooning but not with lobular inflammation. Patients with sarcopenia also had higher prevalence of significant fibrosis (≥F2, 46.0 vs. 25.0%, *p* < 0.001). Sarcopenic patients with NAFLD have a two-fold increased risk for NASH (OR 2.46; 95% CI, 1.35–4.48) and significant fibrosis (OR 2.01; 95% CI, 1.12–3.61). This association persisted after adjustment for classical confounders (age, sex, BMI, and T2DM) but was slightly attenuated after adjusting for HOMA-IR and high-sensitivity C-reactive protein (hs-CRP) which further highlights the role of IR and inflammation as a potential link between the two entities (14).

The association between sarcopenia and the amount of steatosis and fibrosis has been confirmed in a Caucasian population by a recent study by Petta et al. (159) and persisted after adjustment for confounders. The prevalence of sarcopenia gradually increased with the fibrosis stages (fibrosis F0, 22.2%), to F1 (34.9%), to F2 (43.7%), F3 (66.6%) and finally F4 (60.0%) (*p* = 0.002). Moreover, in the presence of sarcopenia, the prevalence of severe fibrosis was higher both in patients with visceral obesity (46.0 vs. 30.9% in non-sarcopenic, *p* = 0.05) and in non-obese patients (44.4 vs. 7.1%, *p* = 0.002). Contrary to the Korean study, in this study, the significant association between sarcopenia and NASH was not maintained after adjusting for demographic and metabolic risk factors (159). These differences highlight not only the heterogeneity in the populations studied (Asian vs. Caucasian) and the definition used, but also suggest that sarcopenia and NAFLD are linked through obesity and IR and probably potentate each other. Indeed, sarcopenia reduces glucose uptake and increases insulin resistance which in turn accelerates fibrosis progression. It is under debate whether these findings should prompt us to include the assessment of sarcopenia in the initial evaluation of patients with NAFLD or vice versa and prospective follow-up studies are mandatory. In line with that, several longitudinal studies support the assumption that sarcopenia is an independent risk factor for the progression of the liver damage and is able to predict clinical outcomes and liver-related mortality.

A large longitudinal cohort study with a 10-year follow-up showed that age-related decrease in ASM and body composition were associated with an increased risk of incident NAFLD, particularly in non-obese subjects. At baseline, individuals with incident NAFLD had higher BMI, FM, and ASM despite a lower ASM adjusted by body weight (ASM-to-weight); during follow-up, they gained more weight, had a lower ASM-to-weight, and had a more important decrease in ASM (43). Another Korean study showed that baseline ASM-to-weight was inversely associated with incident NAFLD and positively associated with the resolution of pre-existing NAFLD. Furthermore, increasing ASM-to-weight had a positive impact both on the risk of incident NAFLD and the resolution of existing NAFLD even after adjusting for metabolic confounders (164). Several longitudinal follow-up studies showed that sarcopenic patients with NAFLD had poor clinical outcomes and increased risk in overall (OR 1.28; 95% CI 1.06–1.5) and specific mortality, particularly cardiovascular, cancer, and diabetes related mortality (162). Another recent study analyzing the NHANES 1999–2004 dataset reported a 78.0% increased risk in all-cause mortality and a 320.0% increase in cardiac-specific mortality in patients with sarcopenia-related NAFLD (128).

Finally, in patients with compensated-advanced chronic liver disease (c-ACLD), sarcopenia is highly prevalent [40.0% in cirrhotic patients (165, 166), and rises up to 70.0% in candidates to liver transplantation—LT] (Supplementary Table 2). Using the Fried Frailty Index, 25.0% of candidates to LT were classified as frail. In patients with cirrhosis, the annual rate of skeletal muscle loss increases with the severity of liver disease from 1.3% in Child A cirrhosis to 6.0% in Child C patients (167). In patients with c-ACLD, both sarcopenia and frailty, significantly increase the overall mortality (more than two-to-five-fold increase) the risk of cirrhosis decompensation (hepatic encephalopathy—HE, ascites), the length of hospital stay (168) and were associated with higher medical costs (169).

Sarcopenia is an independent predictor of mortality in cirrhotic patients even after adjusting for MELD score or the presence of portal hypertension (170). The addition of sarcopenia to the MELD score improved the prediction of short-term survival; The MELD-psoas model outperformed the MELD score and performed similarly to MELD-Na in predicting survival (171). Several studies described a dose-dependent and bidirectional relationship between sarcopenia or frailty and the clinical outcomes in cirrhosis. Thus, a single unit increase in the Fried Frailty Index was associated with a 45.0% increase in mortality on the waiting list for LT (172). Conversely, an increase in HGS by 1.0 kg or the improvement in the gait speed by 0.1 m/s decreased the wait-list mortality by 11.0 and 28.0%, respectively (173). Although sarcopenia and frailty are probably interrelated through a bidirectional relationship, each entity captures different risks and thus explains the different impact on the clinical outcomes in the same patient. Although both sarcopenia and frailty have been largely explored in patients with advanced liver disease, only a minority of studies specifically focused on NAFLD-related cirrhosis (Supplementary Table 2).

The limited number of patients with NAFLD-related cirrhosis, most of whom are coming from LT centers, is mainly due to the fact that NAFLD was for a long time an underrecognized condition and patients were listed both in *U*nited *N*etwork for *O*rgan *S*haring (UNOS) and European Liver Transplant Registry (ELTR) registries as either “cryptogenic cirrhosis” or “other metabolic etiologies” (174). One study reported that patients with NAFLD-related cirrhosis have a 6-fold increased risk of having sarcopenic obesity (44) which in turn is associated with more severe liver disease and worse outcomes (175). These results are not consistent across all studies. For example, one study from Mayo Clinics, found that patients with NAFLD-related cirrhosis listed for LT had a higher prevalence of frailty (49.0%) and myosteatosis (78.0%) and a lower prevalence of sarcopenia (22.0%). The higher prevalence of frailty in patients with NAFLD is probably related to the phenotype of NAFLD candidates to LT (older age, clustering of cardiometabolic comorbidities) which leads to disability, dependency and impaired cognitive function. Frailty is associated with longer hospital stays and increased risk of removal from the waiting list but had no impact on the overall survival after LT (175). The course of sarcopenia following LT is controversial and most of the studies have found little or no improvement probably in a relationship with post-LT complications or immunosuppressive therapy (176, 177).

## Sarcopenic Obesity: A Separate and Different Entity?

Aging is characterized by specific alterations in body composition, particularly by an increase in FM and a decrease in SMM, without evident changes in BMI. The concept of “sarcopenic obesity—SO” was first mentioned by Heber et al. (178) in 1996 and defined as reduced LBM associated with increased FM as determined by BIA. In 2000, Baumgartner (179) described SO in relationship with the decline in physical activity and energy expenditure as the interplay between obesity and sarcopenia defined by DXA. The global prevalence of SO is 11.0% in the general population and rises up to 23.0% in subjects ≥75 years old as reported by a recent meta-analysis (180) but can vary widely depending on the population studied (181, 182).

Situated at the confluence between the actual trends in aging population and the increasing prevalence of obesity, SO is now an emerging health problem responsible for an increased disability in daily activities and reduced quality of life. The morbidity and mortality risk related to SO is greater than risk related to either obesity or sarcopenia alone (183–185). For example, in the British Regional Heart Study, a 6-year prospective study of ≥4,000 men aged 60–70 years, the mortality risk was 55.0% and increased in subjects with both sarcopenia and obesity compared with those with sarcopenia or obesity alone (186). Similar results were found among Japanese Americans elderly men from the Kuakini Honolulu Heart Program (187). It has been shown that compared to patients with sarcopenia or obesity alone, patients with SO are at increased risk of MetS irrespective of ethnicity (Asian, OR 8.28; 95% CI 4.45–15.4, and mixed population Asian and Caucasian, OR 11.59; 95% CI 6.72–19.98, respectively) (188, 189). Several cross-sectional studies also reported an increased risk of hypertension (OR 6.42; 95% CI 4.85–8.48), dyslipidemia (OR 2.82; 95% CI 1.76–4.51) (190), diabetes (OR 2.16; 95% CI 1.08–3.27) (191), or diabetes related complications (OR 6.52; 95% CI 1.47–28.8) (192).

Concerning the cardiovascular risk associated with SO, the results are controversial. Some studies reported an overall increased risk for early atherosclerosis as defined by coronary artery calcifications (OR 1.92; 95% CI 1.16–3.18) (193) and 10-year cardiovascular risk determined by Framingham score (OR 2.49; 95% CI 1.53–4.06) (194) in subjects with SO vs. sarcopenia or obesity alone. Data from the US NHANES database including 11,317 participants also demonstrated that SO was associated with an eight-fold increased risk of cardiovascular disease in both metabolically healthy and unhealthy individuals (195). The British Regional Heart Study, conducted in older men of 60–79 years of age, although found a significant association between SO and cardiovascular mortality, failed to demonstrate an association between SO and coronary heart disease events. Ultimately, the association between SO and individual cardiovascular risk factors—atherogenic lipids profile with low high-density lipoprotein cholesterol (HDL-C) and high triglyceride levels, increased hs-CRP and IR acts together and increases the overall cardiovascular risk and mortality (196).

The risk factors for SO identified by most of the studies are older age (180), sex-related hormonal changes (i.e., postmenopausal increase in visceral fat as a result of low estrogen levels, testosterone deficiency) (197), ethnicity (higher prevalence in Hispanics and non-Hispanic whites compared with non-Hispanics blacks) (181), physical inactivity and clustering of the comorbidities (198). These risk factors are all common among patients with NAFLD and favors SO. The clinical phenotype of NAFLD is typically characterized by older age and clustering of cardiometabolic comorbidities—T2DM, obesity and its complications, and cardiovascular disease—which result in the impairment of the effort capacity and decreased physical activity.

On the other hand, a sizable proportion of patients with NAFLD experienced repetitive restrictive dietary interventions to lose weight which often result in loss of the LBM but gain in FM because of concomitant physical inactivity. Therefore, it is not surprising that the prevalence of SO is higher in patients with NAFLD (ranges from 18.0 to 77.0%) than reported in the general population (144, 158) (Supplementary Table 3). By altering lipid muscle metabolism, IR and the inflammatory pathways, obesity and sarcopenia promote lipotoxicity and not only potentate each other in a kind of vicious circle as discussed above, but also have a negative impact on the natural course of NAFLD both in terms of the evolution of the cardiometabolic conditions and progression of the liver damage. Conversely, the presence of NAFLD significantly impacts the changes in body composition. This has been shown in a small North American weight loss interventional trial which evaluated the relationship between NAFLD and NAFLD resolution and body composition in obese individuals. Despite similar changes in BMI during follow-up, patients with NAFLD had a greater reduction in visceral adipose tissue area (VATA). Furthermore, participants with NAFLD resolution had an even more significant reduction in the VATA while no significant changes occurred in the SMM (199). These findings suggest a possible protective role of FM in preserving muscle mass in weight loss settings but further studies are warranted.

Because of the overlapping in the physiopathological pathways, it is under debate whether the impact of SO on the liver-related outcomes is more than additive. Both sarcopenia and obesity are risk factors for fibrosis progression. As shown in a recent study from the NHANES database from 2017 to 2018 (161), patients with SO had a higher prevalence of significant fibrosis (20.9 vs. 9.4%), and cirrhosis (7.5 vs. 2.6%) than those without these conditions. Even after adjustment for confounders, patients with SO have a two-fold higher risk of having NAFLD-associated significant fibrosis. SO is found in 20.0 to 40.0% of LT candidates. One study has shown that patients with end-stage NAFLD listed for LT have a six-fold increased risk of SO (44).

Both sarcopenia and obesity are established risk factors for the development of hepatocellular carcinoma (HCC) and are predictors for tumor recurrence and overall survival (200, 201). A retrospective study of 465 Japanese patients who underwent liver resection for HCC found that preoperative SO more than doubles the risk of death and HCC recurrence after hepatectomy (202). These results have been confirmed by another Japanese study that showed that patients with SO have a significant decrease in overall and recurrence-free survival at 3- and 5 years despite similar 1-year survival. This study also showed that patients with SO also have more advanced liver lesions with higher rates of multiple tumors, microvascular invasion, moderate differentiation, and satellite nodules (57). A more in-depth analysis of body composition has shown that sarcopenia, intramuscular fat content and visceral adiposity were independent predictors of mortality in patients with HCC. These data emphasize that body composition rather than BMI has significant prognostic value in HCC patients (203).

As most of these data are coming from Asian population with HBV-related cirrhosis, their generalizability to a Caucasian population with NAFLD is limited. Whether the impact of SO on liver and HCC-related outcomes is magnified in the context of the pro-inflammatory and IR milieu which characterizes patients with NAFLD should be determined by future prospective studies.

## Gaps in Knowledge and Future Directions

Sarcopenia and SO are perfect models to illustrate the changes in body composition that occur with aging and result in distinct clinical phenotypes which specifically impact clinical outcomes. Simple clinical tools like BMI or waist circumference (WC) measures are widely used to evaluate nutritional status (204) but are unable to capture the differences in body composition, such as the amount and the distribution of muscle and fat mass. As an example, it is now widely accepted that low BMI is linked to higher mortality rates in relationship with a low SMM and not with a low FM. These data underline the clinical relevance of the body composition and bring into attention a challenging new paradigm also called the “obesity paradox.” This new concept suggests the possible protective role of the FM and reveals the preservation of the lean body mass as an important therapeutic goal (205). This concept is particularly important in patients with NAFLD because weight loss and lifestyle changes have a central role in the management of the disease (206). Preventing lean mass wasting should be a therapeutic goal in patients with NAFLD who are particularly exposed to reduced physical activity and sedentary behavior (207) due to the associated comorbidities. Because a lot of the molecules now tested in randomized clinical trials in NAFLD target multiple metabolic pathways to improve liver damage and some of them are associated with changes in body weight, the assessment of the body composition is now one of the secondary outcomes to assess drug efficacy in the ongoing clinical trials in NAFLD (208). While the assessment of body composition gains more and more recognition in NAFLD clinical trials, its use in routine clinical practice is still limited and warrants increased awareness of the clinical practitioners.

The assessment of body composition has been extensively investigated in sarcopenia and SO aiming to improve the diagnosis of muscle disease in many settings. However, this triggered a plethora of various diagnostic tools and different cut-offs which resulted either in underestimating or overestimating the prevalence of these conditions according to the populations studied (209). The cut-off that is better correlated with clinical outcomes has to be determined by future longitudinal prospective follow-up studies for each diagnostic tool. Among the diagnostic methods employed to assess muscle mass and quality (210), some of them (CT, MRI) can also be performed to evaluate the presence of hepatic steatosis which makes possible a single examination for both conditions.

Another gap in the knowledge with important clinical implications is to distinguish between each of (1) the quantitative assessment of the SMM and body composition resulting into sarcopenia or SO (2) assessment of the muscle strength resulting in functional impairment and frailty and (3) assessment of the muscle composition resulting in myosteatosis. A better understanding of the clinical and prognostic information brought by each of these measures will allow for more individualized recommendations to use one test or another depending upon the clinical phenotype and management strategies used for each patient.

Although the association between sarcopenia and NAFLD is largely supported by the existing literature, there is insufficient evidence to argue for a direct causal relationship between these two entities beyond the common pathophysiological pathways. In a kind of an “egg and chicken story” it is unclear whether sarcopenia is coming first and represents a risk factor for NASH progression, or it is rather a complication of NAFLD which occurs later along with the worsening of the liver damage (Figure 2). The studies published to date suggest that the two entities are interconnected through a bidirectional relationship and each one increases the risk for the other, in some kind of a vicious circle. Thus, the crosstalk between muscle, liver and adipose tissue plays a central role in shaping the body composition and defines a new clinical phenotype concept called *sarcopenic NAFLD*, which might account for the heterogeneity of the NAFLD phenotypes. This concept is partially supported by clinical and experimental data suggesting that sarcopenia and NAFLD are both consequences of lipotoxicity and ectopic lipid storage in skeletal muscle (myosteatosis) and hepatocytes. Yet, it has to be determined whether screening for sarcopenia should be implemented in routine clinical practice in patients with NAFLD. Hypothetically, screening for sarcopenia and assessing muscle composition in NAFLD would allow to better stratify patients according to the clinical phenotype and identify those at higher risk of disease progression and severe clinical outcomes. A first line screening for sarcopenia could be easily done in clinical practice using available questionnaires such as GLIM criteria (*Global Leadership Initiative on Malnutrition*) (211), a worldwide consensus for categorizing the various forms of malnutrition based on phenotypic (body morphologies) and etiologic (food intake and disease burden) criteria. European Association for the Study of the Liver (EASL) guidelines clearly state that obesity does not rule out malnutrition and recommend to perform a rapid nutritional screen for sarcopenia in all patients with cirrhosis and to complete a detailed assessment in those at high risk of malnutrition (139).

Finally, in patients with NAFLD, a more subtle analysis of the clinical phenotypes would potentially lower the heterogeneity of the population included in NAFLD clinical trials, thus increasing the chances to prove drugs efficacy. This would be particularly helpful in the actual landscape of drug development in NAFLD with a lot of molecules that failed to prove their efficacy despite promising results in experimental and preliminary phase I and II clinical trials. On the other hand, sarcopenia is a modifiable risk factor through lifestyle interventions. Thus, the identification of *sarcopenic NAFLD* phenotype will allow a better counseling of the nutritional interventions with a focus on diet composition and physical exercise to avoid skeletal muscle loss and weight regain which is also known as the “accordion effect” (137). Subtle changes in body composition might help to restore homeostasis in the muscle-liver-adipose tissue axis and promote a long-term sustained weight loss which is a key to modify the natural course of NAFLD.

## Author Contributions

RP designed, wrote, and revised the manuscript. CS and VZ equally contributed to writing the manuscript. HS performed literature research and entered data. TM and VR critically revised the manuscript. All authors approved the final version of the manuscript.

## Conflict of Interest

The authors declare that the research was conducted in the absence of any commercial or financial relationships that could be construed as a potential conflict of interest.

## Publisher's Note

All claims expressed in this article are solely those of the authors and do not necessarily represent those of their affiliated organizations, or those of the publisher, the editors and the reviewers. Any product that may be evaluated in this article, or claim that may be made by its manufacturer, is not guaranteed or endorsed by the publisher.
