# Supplementary material for: Impact of Sarcopenia on the Severity of the Liver Damage in Patients With Non-alcoholic Fatty Liver Disease
Source: Front Nutr. 2022 Jan 17;8:774030. doi: 10.3389/fnut.2021.774030 (PMC8802760; doi:10.3389/fnut.2021.774030)
Supplement: Supplementary file 1 [file Data_Sheet_1.docx]

**Supplementary material**

**Table 1 –** Overview of representative clinical studies on associations between sarcopenia and non-alcoholic fatty liver disease (NAFLD) spectrum .

| **Study design, Country (ethnicity)** | **Author, year [ref]** | **Study population** | **Method to diagnosis liver disease** | **Method to diagnosis sarcopenia** | **Sarcopenia prevalence** | **Outcomes** |
| --- | --- | --- | --- | --- | --- | --- |
|  |  |  |  |  |  |  |
| Meta-analysis  (Asia and Europe) | Yu et al., 2018 (1) | n=3,226 among 3 studies associating sarcopenia with NASH and liver fibrosis of NAFLD | **Histology by biopsy**,  and **predictive non-invasive scores** | **DXA** and **BIA** | NA | Sarcopenia is associated with the severity of NAFLD, respectively, [OR=2.35, 95% CI= 1.45 – 3.81] for NASH and [OR=2.41, 95% CI= 1.94 – 2.98] for advanced liver fibrosis |
| Meta-analysis  (Korea, and United States) | Wijarnpreecha et al., 2018 (2) | n=27,804 among 5 studies in patients with or without sarcopenia and NAFLD risk | **US**, **CT scan**, **predictive non-invasive scores**, and **histology by biopsy** | **DXA**, **MRI**, and **BIA** | NA | Sarcopenic patients have 1.5-fold increased risk of NAFLD than those without sarcopenia [OR=1.54, 95% CI= 1.05 – 2.26] |
| Meta-analysis  (Korea, Japan, and Italy) | Pan et al., 2018 (3) | n=19,024 among 6 studies with sarcopenic and non-sarcopenic individuals | **CT scan, predictive non-invasive scores**, **histology by biopsy**, and **FibroScan®** | **DXA** and **BIA** | NA | Sarcopenia is associated with 1.3-fold increased NAFLD risk [OR=1.29, 95% CI= 1.12 – 1.49], and 1.6-fold more significant fibrosis NAFLD-related [OR=1.57, 95% CI= 1.29 – 1.90] |
| Meta-analysis  (China, Japan, Korea, Italy, Belgium, and America) | Cai et al., 2020 (4) | n>48,000 among 19 studies including NAFLD and non-NAFLD patients | **US**, **FLI**, **HSI**, **CT scan**, **predictive non-invasive scores**, **histology by biopsy**, and **FibroScan®** | **DXA, BIA**, and **CT** | NA | NAFLD patients have 1.77-fold lower SMI values than those without NAFLD [OR=1.77, 95% CI= 1.15 – 2.39], and sarcopenic individuals present higher occurrence of NAFLD [OR=1.33, 95% CI= 1.20 – 1.48], NASH [OR=2.42, 95% CI= 1.27 – 3.57], and NAFLD-related significant fibrosis [OR=1.56, 95% CI= 1.34 – 1.78] |
| Retrospective cross-sectional study  (Korea) | Moon et al., 2013 (5) | n=9,565 subjects, those 1,848 with NAFLD (FLI ≥ 60.0) | **Fatty liver index** (FLI)  Low FLI < 20.0  Intermediate FLI ≥ 20.0 and < 60.0  High FLI ≥ 60.0 | **BIA –** SMM to VFA ratio  (SVR, g/cm²)  Lowest quartile ≤ 230.0  Highest quartile > 335.0  **BIA –** SMI (%)  Values not informed | NA | SMM is inverse correlated with VFA, and higher SVR is associated with low NAFLD incidence |
| Prospective cross-sectional study  (Korea) | Hong et al., 2014 (6) | n=452 apparently healthy adults | **CT** – liver attenuation index (LAI) < 5.0 HU | **DXA** – SMI (%)  M < 39.8  W < 34.1 | n=128 (28.3%)  M: 32 (19.2%)  W: 96 (33.7%) | Lower muscle mass increased NAFLD risk [OR=5.88, 95% CI= 2.33 – 14.84] |
| Prospective cross-sectional study  (Japan) | Hashimoto et al., 2016 (7) | n=145 patients with type 2 diabetes, of whom 97 with NAFLD | **FibroScan® – Controlled attenuation parameter**  (CAP, dm^-1^) > 237.8  and **FIB-4** ≥ 2.67 | **BIA** – SMI (%)  Values not informed | NA | SMI is independently correlated with hepatic steatosis in men with T2DM, but not in women |
| Retrospective cross-sectional study  (Korea) | Lee et al., 2016 (8) | n=2,761 patients with NAFLD | **Predictive non-invasive scores**  (FIB-4 ≥ 2.67, NFS, and Forns index) | **DXA** – ASM-to-BMI (m²)  M < 0.789  W < 0.521 | n=337 (12.2%)  M: 132 (10.6%)  W: 205 (13.5%) | Sarcopenia is associated with two-fold increased risk of significant liver fibrosis, independently of liver enzyme levels, IR or obesity |
| Retrospective cross-sectional study  (Korea) | Kim et al., 2016 (9) | n=3,739 subjects, those 389 with NAFLD (FLI ≥ 60.0) | **Fatty liver index** (FLI)  NAFLD if FLI ≥ 60.0 | **DXA –** ASM-to-weight (%)  Values not informed | NA | NAFLD is negatively correlated with ASM-to-weight for both genders, respectively for men [OR=1.35, 95% CI= 1.17 – 1.54], and for women [OR=1.36, 95% CI= 1.18 – 1.55] |
| Prospective cross-sectional study  (Korea) | Koo et al., 2017 (10) | n=309 participants, those 117 with NAFL, and 123 with NASH | **Histology by biopsy** ≥ 5.0% macrovesicular steatosis  **Stiffness by FibroScan** | **BIA**   1. ASM-to-weight (%)   M < 29.0  W < 22.9   1. ASM-to-BMI (m²)   M < 0.789  W < 0.512 | **Criteria a:**  NAFL:  n=21 (17.9%)  NASH:  n=43 (35.0%)  **Criteria b:**  NAFL:  n=17 (14.5%)  NASH:  n=33 (26.8%) | Sarcopenia is associated with 2-fold increased NASH risk and significant liver fibrosis |
| Prospective longitudinal study  (Korea) | Kim et al., 2018 (11) | n=15,567 participants, those 2,943 with NAFLD at baseline | Hepatic steatosis index  **HSI** > 36.0 | **BIA** – ASM-to-weight (%)  Lowest sex-specific tertiles: values not informed | **Without NAFLD:**  n=4207 (33.3%)  **With NAFLD:**  n=980 (33.3%) | ASM-to-weight is inversely associated with NAFLD development and positively associated with resolution of existing NAFLD |
| Prospective cross-sectional study  (China) | Zhai et al., 2018 (12) | n=494 elderly patients (60 to 96 years old), those 158 with sarcopenia | **Abdominal US** associated with not significant alcohol consumption (≥20g/d) | **DXA** – ASMI (Kg/m²)  M < 7.0  W < 5.4  **Handgrip Strength** (HGS, Kg)  M < 26.0  W < 18.0  **Usual gait speed** (m/s)  6-m < 0.8 | **Without NAFLD:**  n=158 (31.9%)  M: 87 (40.3%)  W: 71 (25.5%)  **With NAFLD:**  n=35 (22.15%) | NAFLD is not independently associated with sarcopenia |
| Retrospective cross-sectional study  (USA) | Wijarnpreecha et al., 2019 (13) | n=11,325 participants apparently healthy | **Abdominal US** and **NAFLD fibrosis**  **score** (NFS) | **BIA** – SMI (%)  M ≤ 37.0  W ≤ 28.0 | **NAFLD *vs*. non-NAFLD:**  n=46.7% *vs*. 27.5%  **With NAFLD-associated advanced fibrosis**:  n= 7.8% *vs*. 1.6% | Sarcopenia is associated with 2.3-fold higher risk of NAFLD [OR=2.31, 95% CI= 2.01 – 2.64], and 1.79-fold higher risk of NAFLD-associated advanced fibrosis [OR=1.79, 95% CI= 1.18 – 2.72], independently of metabolic factors |
| Retrospective cross-sectional study  (USA) | Peng et al., 2019 (14) | n=2,551 elderly patients (60 to 75 years old) apparently healthy | **Abdominal US** | **BIA** – SMM-to-height² (Kg/m²)  M < 10.76  W < 6.75  SMI (%)  M < 37.0  W < 28.0  **Gait speed** (m/s)  ≤ 0.8 | SMM-to-height² and gait speed criteria:  n=759 (29.7%)  SMI and gait speed criteria:  n=985 (38.6%) | Severe hepatic steatosis is associated with an increased risk of sarcopenia by SMI criteria [OR=1.73, 95% CI= 1.18 – 2.72] |
| Retrospective cross-sectional study  (USA) | Golabi et al., 2020 (15) | n=4,611 participants, those 1,351 with NAFLD | **US Fatty liver index** (FLI)  NAFLD if FLI ≥ 60.0  **High fibrosis risk by predictive non-invasive scores:**  NFS > 0.676 and  FIB-4 > 2.67 | **DXA** – ASM-to-BMI (m²)  M < 0.789  W < 0.512 | **Without NAFLD:**  n= 183 (5.6%)  **With NAFLD:**  n=239 (17.7%)  M: 14.3%  W: 18.9% | NAFLD individuals have 2.9-fold higher sarcopenia than those without NAFLD |

**Legend:** NASH, non-alcoholic steatohepatitis; NAFLD, non-alcoholic fatty liver disease; M, men; W, women; DXA, dual-energy X-ray absorptiometry; BIA, bioimpedance analysis; US, ultrasound; CT, computed tomography; MRI, magnetic resonance imaging; BMI, body mass index; SMM, skeletal muscle mass; VFA, visceral fat area = visceral adipose tissue area at the level of the umbilicus; SVR, skeletal muscle mass adjusted to visceral fat area; SMI, skeletal muscle index; ASM, appendicular skeletal mass (sum of muscle mass in Kg of both arms and legs); ASMI, appendicular skeletal mass adjusted to height²; ASM-to-BMI, appendicular skeletal mass adjusted to BMI; ASM-to-weight, appendicular skeletal mass adjusted to body weight; SMM-to-height², skeletal muscle mass adjusted to height²; HGS, Handgrip Strength; IR, insulin resistance; T2DM, type 2 diabetes; FibroScan®, transient hepatic elastography; LAI, liver attenuation index; CAP, controlled attenuation parameter; NFS, NAFLD fibrosis score; FIB-4, fibrosis-4-index; HSI, hepatic steatosis index; FLI, Fatty liver index; NA, not applicable.

**Table 2 –** Overview of representative clinical studies on associations between sarcopenia and ESLD.

| **Study design, Country (ethnicity)** | **Author, year [ref]** | **Study population** | **Method to diagnosis liver disease** | **Method to diagnosis sarcopenia** | **Method to diagnosis obesity** | **Sarcopenia prevalence** | **SO prevalence** | **Outcome** |
| --- | --- | --- | --- | --- | --- | --- | --- | --- |
| Retrospective longitudinal study (Canada) | Tandon et al., 2012 (16) | n=142 patients with cirrhosis listed for LT, those 11 due to NASH etiology | **MELD score**  **Child-Pugh classification** | **MRI & CT**  SMA at L3-to-height² = SMI (cm²/m²)  M ≤ 52.4  W ≤ 38.5 | NA | n=58 (41.0%)  M: 46 (54.0%)  W: 12 (21.0%) | NA | Sarcopenia is an independent predictor of increased mortality on awaiting-LT |
| Retrospective longitudinal study (USA) | Cruz et al., 2013 (17) | n=234 patients candidates to LT, those 28 due to NASH etiology | **MELD score** | **CT** – SMA at L3-L4 to height²= SMI (cm²/m²)  M ≤ 52.4  W ≤ 38.5 | **BMI** (Kg/m²)  ≥ 30.0 and  **CT** – Visceral and subcutaneous fat: values not informed | n=164 (70.0%) | n=41 (56.0%) | NASH patients present higher amounts of visceral fat associated with less muscle mass than other types of liver diseases |
| Retrospective cross-sectional study (USA) | Issa et al., 2014 (18) | n=75 patients, those 25 with NASH, and 25 with NASH-cirrhosis | **Histology by biopsy** | **CT** – SMA at L4-to-height²= SMI (cm²/m²)  Values not informed | NA | NA | NA | Sarcopenia is associated with NASH and NASH-related cirrhosis, worsening the muscle mass with the cirrhosis progression |
| Retrospective longitudinal study (Canada) | Montano-Loza et al., 2014 (19) | n=248 patients with cirrhosis underwent LT, those 14 with NASH etiology | **MELD score**  **Child-Pugh classification** | **CT** – SMA at L3-to-height²= SMI (cm²/m²)  **If BMI < 25.0Kg/m²:**  M & W ≤ 43.0  **If BMI ≥ 25.0Kg/m²:**  M ≤ 53.0  W ≤ 41.0 | NA | n=112 (45.0%)  **With NASH:**  n=10 (9.0%) | NA | Sarcopenia is a predictive condition of longer hospital stays and higher risk of bacterial infections after LT |
| Retrospective longitudinal study (USA) | Carias et al., 2016 (20) | n=207 patients with cirrhosis undergoing LT, those 45 due to NASH etiology | **MELD score** | **CT** – SMA at L3-to-height² = SMI (cm²/m²)  M ≤ 52.4  W ≤ 38.5 | **BMI** (Kg/m²)  ≥ 30.0 | n=122 (59.0%) | n=86 (41.7%) | NASH is associated with six-fold increased risk of SO in cirrhotic patients awaiting-LT [OR=6.03, 95% CI= 1.44 – 25.27] |
| Retrospective longitudinal study (USA) | Carey et al., 2017 (21) | n=396 patients with ESLD awaiting LT, those 42 due to NASH etiology | **MELD score** | **CT** – SMA at L3-to-height² = SMI (cm²/m²)  M < 50.0  W < 39.0 | NA | n=178 (45.0%)  M: 139 (50.2%)  W: 39 (33.0%)  **With NASH:**  n=20 (11.0%) | NA | Lower SMI is associated with mortality in patients with ESLD waiting-LT |
| Retrospective longitudinal study (Italy) | Begini et al., 2017 (22) | n=92 patients with HCC, those 19 due to NASH etiology | **Barcelona Clinic Liver Cancer (BCLC) classification** | **CT** – SMA at L3-to-height² = SMI (cm²/m²)  **If BMI < 25.0Kg/m²:**  M & W ≤ 43.0  **If BMI ≥ 25.0Kg/m²:**  M ≤ 53.0  W ≤ 41.0 | NA | n=37 (40.2%)  **With NASH:**  n=8 (21.6%) | NA | Sarcopenia is a predictor of reduced overall survival in patients with HCC [OR=2.37, 95% CI= 1.28 – 4.39] |
| Retrospective longitudinal study (France) | Golse et al., 2017 (23) | n=256 patients with cirrhosis undergoing LT, those 5 due to NASH etiology | **Histology by biopsy** | **CT** – PMA-to-height² (mm²)  M ≤ 1561.0  W ≤ 1464.0 | NA | n=57 (22.0%)  M: 24 (42.0%)  W: 33 (58.0%) | NA | Sarcopenia is the most important predictive factor impacting negatively 1-year survival after LT [OR=17.62, 95% CI= 6.04 – 51.37] |
| Retrospective longitudinal study (USA) | Montano-Loza et al., 2018 (24) | n=678 patients with cirrhosis assessed for LT, those 152 due to NASH etiology | **MELD score** | **CT** – Criteria not assessed | **CT** – VATI (cm²/m²)  ≥ 65.0 | NA | n=292 (43.0%)  M: 206 (45.1%)  W: 86 (38.9%)  **With NASH:**  M: 43 (21.0%) | VATI ≥65 cm²/m² is associated with five-fold increased risk of HCC recurrence in male cirrhotics undergoing LT |
| Retrospective longitudinal study (Canada) | Bhanji et al., 2018 (25) | n=675 patients with cirrhosis, those 154 due to NASH etiology | Presence of **acute confusional syndrome** and/or patients taking **lactulose/rifaximin** | **CT** – SMA at L3-to-height² = SMI (cm²/m²)  M ≤ 50.0  W ≤ 39.0  **CT** – Myosteatosis  If BMI < 24.9 Kg/m²: HU < 41.0  If BMI ≥ 25.0 Kg/m²: HU < 33.0 | NA | n=242 (35.8%) | NA | Myosteatosis and sarcopenia are independent factors for overt HE in cirrhotic patients |
| Retrospective longitudinal study (Australia) | Vidot et al. 2019 (26) | n=205 patients with cirrhosis assessed for LT, those 14 due to NASH etiology | **Liver-specific subjective global assessment** (SGA), **Child Pugh classification** and **MELD score** | **CT – Corrected total psoas muscle area at L3**  (cTPA, mm²/m²)  M ≤ 545.0  W≤ 385.0 | **BMI** (Kg/m²)  ≥ 30.0 | **All patients:**  n=178 (86.8%)  **With NASH:**  n= 11 (6.0%)  **Non-obese:**  M: 63.0%  W: 23.0% | n= 89.0%  M: 73.0%  W: 16.0% | Muscle wasting is associated with increased severity of the liver disease in patients waiting-LT |

**Legend:** NASH, non-alcoholic steatohepatitis; NAFLD, non-alcoholic fatty liver disease; ESLD, end-stage liver disease; MELD, Model for End-Stage Liver Disease; LT, liver transplantation; HCC, hepatocellular carcinoma; HE, hepatic encephalopathy; M, men; W, women; SO, sarcopenic obesity; CT, computed tomography; MRI, magnetic resonance imaging; SGA, liver-specific subjective global assessment; BMI, body mass index; SMI, skeletal muscle index; SMA-L3-or-L4, sectional muscle area at the level of the 3^rd^ or 4^th^ lumbar vertebra; SMI-L3-or-L4, skeletal muscle index (SMA adjusted by height²); PMA-to-height², psoas muscle area adjusted to height²; cTPA, corrected total psoas muscle area at L3; VATI, visceral adipose tissue index; NA, not applicable.

**Table 3 –** Overview of representative clinical studies on associations between sarcopenic obesity (SO) and non-alcoholic fatty liver disease (NAFLD) spectrum.

| **Study design, Country (ethnicity)** | **Author, year [ref]** | **Study population** | **Method to diagnosis liver disease** | **Method to diagnosis sarcopenia** | **Method to diagnosis obesity** | **Sarcopenia prevalence** | **SO prevalence** | **Outcome** |
| --- | --- | --- | --- | --- | --- | --- | --- | --- |
| Prospective longitudinal study (USA) | Dasarathy et al., 2014 (27) | n=148 patients with NAFLD | **Histology by biopsy** | **BIA** – FFM (Kg)  Values not informed  **CT** – SMA at L4-to-height²= SMI (cm²/m²)  Values not informed | **BIA** – FM (Kg)  Values not informed  **CT – Visceral adipose tissue area**  (VAT, cm²)  Values not informed | NA | NA | Total fat-to-muscle area is higher in patients with NASH than with steatosis, suggesting that SO is more severe in NASH individuals |
| Retrospective cross-sectional study (Korea) | Lee et al., 2015 (28) | n=15,132 participants | Predictive non-invasive scores **(HSI, CNS, LFS, BARD, and FIB-4)** | **DXA** – ASM-to-weight (%)  M < 32.2  W < 25.5 | **BMI** (Kg/m²)  ≥ 25.0 | n=2004 (13.5%)  M: 697 (34.8%)  W: 1307 (65.2%)  **With steatosis by:**  HIS, n=178 (9.0%)  CNS, n=419 (25.0%)  LFS, n=609 (30.0%) | n=2455 (16.2%)  M: 876 (35.7%)  W: 1579 (64.3%)  **With steatosis by:**  HIS, n=1592 (65.0%)  CNS, n=1728 (83.0%)  LFS, n=1490 (61.0%) | Sarcopenia is associated with increased NAFLD risk and advanced fibrosis |
| Retrospective cross-sectional study (USA) | Shen et al., 2016 (29) | n=9,985 participants | **Abdominal US** | **BIA** – ASMI (Kg/m²)  M ≤ 10.75  W ≤ 6.75 | **Body fat** (%)  M ≥ 27.0  W ≥ 38.0 | n=2,018 (20.2%) | n=1,542 (15.4%) | Sarcopenia is not associated with NAFLD prevalence [OR=1.00, 95% CI= 0.79 – 1.27] |
| Prospective longitudinal study (Italy) | Petta et al., 2017 (30) | n=225 patients with NAFLD | **Histology by biopsy**  ≥ 5.0% macrovesicular steatosis | **BIA** – ASM-to-weight (%)  M ≤ 37.0  W ≤ 28.0  **Phase Angle** (PA, °) < 5.4 | **WC** (cm)  M ≥ 102.0  W ≥ 88.0 | **PA criteria:**  n=13 (5.8%)  **ASM-to-weight criteria:**  n=98 (43.6%), of those 4/65 (6.1%) non-obese  **F0-F2:**  n=22 (14.2%)  **F3-F4:**  n=27 (38.0%) | 41/160 (25.6%) among obese subjects  Severe fibrosis:  n=19/41 (46.0%) | Sarcopenia have a linear increase with severity of liver fibrosis and steatosis |
| Retrospective longitudinal study  (USA) | Rachakonda et al., 2017 (31) | n=129 individuals class II/III obesity  (BMI ≥ 35.0 Kg/m²), those 58 with NAFLD | **Abdominal CT –**  Liver-spleen attenuation ratio  (L:S ratio)  **NAFLD presence**, if L:S < 1.1  **NALD resolution**, if L:S ≥ 1.1 | **DXA** – FFM (Kg)  Values not informed | **DXA** – FM (Kg)  Values not informed  **CT – Visceral adipose tissue area**  (VAT, cm²)  Values not informed | NA | NA | NAFLD is associated with higher VAT, but not with sarcopenia |
| Retrospective cross-sectional study (Korea) | Choe et al., 2018 (32) | n=1,828 outpatients, those 716 with NAFLD | **Abdominal CT & US** | **CT** – SMA at L3-to-BMI (cm/(Kg/m²))  Mild sarcopenia:  M ≤ 8.37  W ≤ 7.47  Severe sarcopenia:  M ≤ 7.04  W ≤ 6.12 | **BMI** (Kg/m²)  ≥ 25.0 | n=454 (24.8%), of those 270/1341 (20.1%) non-obese  in NAFLD presence:  n=97 (35.9%) | n=184/487 (37.8%)  in NAFLD presence:  n=141 (76.6%) | NAFLD risk increases according to the severity of sarcopenia |
| Retrospective longitudinal study (Korea) | Lee et al., 2019 (33) | n=4,398 subjects without NAFLD at baseline, whom 591 developed NAFLD at 10-year follow-up | **Abdominal US** | **BIA** – Δ ASM (Kg)  M ≤ -1.13  W ≤ -1.32 | **BMI** (Kg/m²)  ≥ 25.0  **Δ FM** (Kg)  M ≥ 2.91  W ≥ 3.61 | Non-obese subjects with NAFLD:  n=140 (11.3%) | Obese subjects with NAFLD:  n=56 (24.2%) | Progressive increase in FM and decrease in muscle mass are significantly associated with NAFLD incidence, especially in women |
| Prospective cross-sectional study (European) | Alferink et al., 2019 (34) | n=4,609 participants, of whom 1,623 with NAFLD | **Abdominal US** and  **FibroScan® –** Liver stiffness measurements  (LSM, KPa)  NASH or advanced NAFLD: LSM ≥ 8.0 | **DXA** – ASMI (Kg/m²)  M ≤ 7.25  W ≤ 5.67  **Handgrip Strength**  (HGS, Kg)  **M**:  If BMI ≤24.0, HGS ≤ 29.0  If BMI ≥24.1 to ≤ 28.0, HGS ≤ 30.0  If BMI >28.0 , HGS ≤ 32.0  **W**:  If BMI ≤23.0, HGS ≤ 17.0  If BMI ≥23.1 to ≤ 26.0, HGS ≤ 17.3  If BMI ≥26.1 to ≤ 29.0, HGS ≤ 18.0  If BMI >29.0, HGS ≤ 21.0  **Gait speed** (m/s)  **M**:  If height ≤ 173.0, < 0.65  If height > 173.0, < 0.76  **W**:  If height ≤ 159.0, < 0.65  If height > 159.0, < 0.76 | **BMI** (Kg/m²)  ≥ 25.0  **Android-to-gynoid fat ratio** (AGR): values not informed | **All patients**  **Pre-sarcopenia:**  n=271 (5.9%)  **Sarcopenia:**  n=207 (4.5%)  **NAFLD presence**  **Pre-sarcopenia**:  M: 14/67 (20.9%)  W: 14/94 (14.9%)  **Sarcopenia**:  M: 14/67 (20.9%)  W: 8/94 (8.5%) | **NAFLD presence**  **Pre-SO:**  M: 13/659 (2.0%)  W: 56/803 (0.7%)  **SO:**  M: 22/659 (3.3%)  W: 4/803 (0.5%) | Fat mass distribution (AGR) is a better predictor for NAFLD than lean body mass |
| Retrospective cross-sectional study (Korea) | Kang et al., 2020 (35) | n=13,502 individuals, those 2,092 with NAFLD | **Steatosis:**  HSI > 36.0  **Advanced fibrosis:**  FIB-4 ≥ 1.30  BARD score ≥ 2.0 | **Handgrip Strength**  (HGS-to-BMI, m²)  Lowest quartile < 0.90 | **BMI** (Kg/m²)  ≥ 25.0  **WC** (cm)  M ≥ 90.0  W ≥ 85.0 | n=3,193 (23.6%)  **NAFLD presence:**  n=45.0%  **Advanced fibrosis:**  **FIB-4**, n=18.0%  **BARD**, n=63.9% | n=1,915 (14.2%) | Low muscle strength is associated with NAFLD [OR=3.62, 95% CI= 3.25 – 4.03] and increased risk of advanced fibrosis [OR=2.29, 95% CI= 1.68 – 3.13] |
| Retrospective cross-sectional study (China) | Gan et al., 2020 (36) | n=3,536 participants, those 1,088 with NAFLD | **Abdominal US** | **DXA** – ASM-to-weight (%)  M < 28.6  W < 24.1  **Handgrip Strength**  (HGS-to-weight, %)  M < 51.3  W < 35.4 | **BMI** (Kg/m²)  ≥ 25.0  **WC** (cm)  M ≥ 90.0  W ≥ 80.0 | **Without NAFLD:**  n=119 (4.9%)  **NAFLD:**  n=246 (22.6%) | **Without NAFLD:**  By BM, n=51 (2.1%)  By WC, n=89 (3.6%)  **NAFLD:**  By BM, n=201 (18.5%)  By WC, n=231 (21.2%) | Low muscle mass, low muscle strength, sarcopenia, and SO are associated with NAFLD |
| Retrospective cross-sectional study (European) | De Munk et al., 2021 (37) | n=45 patients with NAFLD, those 18 with liver fibrosis | **MRI** – Hepatic fat fraction > 5.0% and  **FibroScan® –** Liver stiffness measurements  (LSM, KPa)  F2-F3: LSM ≥ 7.0 | **DXA** – ASMI (Kg/m²)  M ≤ 7.25  W ≤ 5.67  **Handgrip Strength**  (HGS, Kg)  If BMI >28.0, HGS ≤ 32.0  **W**:  If BMI ≥23.1 to ≤ 26.0, HGS ≤ 17.3  If BMI ≥26.1 to ≤ 29.0, HGS ≤ 18.0  If BMI >29.0, HGS ≤ 21.0 | **BMI** (Kg/m²)  ≥ 25.0  **FMI** (Kg/m²) and **Body fat** (%): values not informed | DXA criteria:  n=0 (0.0%)  HGS criteria:  n=2 (4.4%) | NA | No correlation between myosteatosis and the degree of hepatic steatosis or fibrosis, but with HOMA-IR and body fat measurements |
| Prospective cross-sectional study (Italy) | Pacifico et al., 2020 (38) | n=234 overweight and obese youths, those 95 with NAFLD | **Abdominal US** and **histology by biopsy** | **DXA** – ASM-to-weight (%) and RMM (%)  Lowest tertiles: values not informed | **BMI** (Kg/m²)  ≥ 85.0^th^ percentile age- and gender-specifics | NA | RMM criteria:  n=78 (33.3%)  M: 38 (28.8%)  W: 40 (39.2%)  **NAFLD presence:**  n=43 (18.4%) | Children in the lowest tertile of RMM present an increased risk of NAFLD [OR=2.80, 95% CI= 1.57 – 5.02], and higher prevalence of NASH, than those in the other two tertiles (70.8% *vs*. 29.2) |
| Retrospective cross-sectional study (China) | Wang et al., 2021 (39) | n=578 individuals, those 154 with NAFLD | **Abdominal US** | **DXA** – ASMI (Kg/m²)  M ≤ 7.0  W ≤ 5.4  **Handgrip Strength**  (HGS, Kg)  M < 26.0  W < 18.0  **Gait speed** (m/s) < 0.8 | **BMI** (Kg/m²)  ≥ 25.0 | **Without NAFLD:**  M: 6/62 (9.7%)  W: 29/362 (8.0%)  **With NAFLD:**  M: 6/30 (20.0%)  W: 19/124(15.3%) | **Without NAFLD:**  M: 2/62 (3.2%)  W: 3/362 (0.8%)  **With NAFLD:**  M: 3/30 (10.0%)  W: 4/124 (3.2%) | Sarcopenia and SO lead to an increased risk of NAFLD, mostly predicted by lower muscle mass, than by lower muscle strength |
| Retrospective cross-sectional study  (USA) | Wijarnpreecha et al., 2021 (40) | n=1,925 participants, those 31.0% to 41.8% with NAFLD, depending in the criteria | **FibroScan® – LSM:**  ≥F2, LSM ≥ 8.0  ≥F4, LSM ≥ 13.1  **CAP** ≥ 263 or **CAP** ≥ 285 | **DXA –** ASM-to-BMI (m²)  M < 0.789  W < 0.512 | **Body fat** (%)  M ≥ 25.0  W ≥ 35.0 | NA | n=194 (7.8%)  **NAFLD presence:**  By CAP≥263, n=69.7%  By CAP≥285, n=62.5% | SO is independently associated with an increased risk of NAFLD and NAFLD-associated with significant fibrosis |
| Retrospective cross-sectional study  (Korea) | Kang et al., 2021 (41) | n=178 patients with NAFLD | **Histology by biopsy** | **CT** – SMA at L3-to-height² = SMI (cm²/m²)  M < 50.0  W < 39.0  **Body composition area** (cm²/m²) | **BMI** (Kg/m²)  ≥ 25.0  **SATI** (cm²/m²)  M ≥ 65.5  W ≥ 77.1  **VATI** (cm²/m²)  M ≥ 93.9  W ≥ 64.0 | NA | NA | Unlike SMI and SATI, the VATI is positively correlated with severe NASH [OR=8.66, 95% CI= 2.13 – 46.40], and an independent risk factor for advanced fibrosis [OR=6.77, 95% CI= 1.81 – 29.90] |
| Retrospective Longitudinal study (Belgium) | Nachit et al., 2021 (42) | n=184 obese patients, those 150 with NAFLD (36 NAFL and 114 NASH) | **Histology by biopsy** and **NAS score** | **CT** – SMA at L4-to-height² = SMI (cm²/m²)  M ≤ 50.0  W ≤ 39.0  **BIA** – ASM-to-weight (%)  Class I sarcopenia:  M < 37.0  W < 27.6  Class II sarcopenia:  M < 31.5  W < 22.1  **BIA** – ASMI (Kg/m²)  Moderate sarcopenia:  M > 8.51 to < 10.75  W > 5.76 to < 6.75  Severe sarcopenia:  M ≤ 8.50  W ≤ 5.75 | **BMI** (Kg/m²)  ≥ 30.0 | NA | n=8 (4.3%)  **NAFL presence:**  n=1 (2.8%)  **NASH presence:**  n=1 (0.9%) | Muscle fat, but not muscle mass, is strongly and independently associated with NASH |
| Retrospective longitudinal study  (UK) | Linge et al., 2021 (43) | n=5,326 participants, those 1,204 with NAFLD | **MRI –** Liver proton density fat fraction (PDFF) > 5.0% and lack of excess alcohol consumption  **Predictive non-invasive scores** (AST:ALT ratio; FIB-4; NAFLD fibrosis score) | **MRI –** Fat-tissue free muscle volume (FFMV) < 25^th^ percentile adjusted by height² (L/m²)  M < 3.6  W < 3.0  **DXA –** ASMI (Kg/m²)  M < 7.0  W < 6.0  **Handgrip Strength**  (HGS, Kg)  M < 27.0  W < 16.0  **Physical performance:** self-reported  (slow walking pace; no stair climbing; and ≥ 1 fall in the past year) | **MRI –** Muscle-fat infiltration (MFI, %)  > 75^th^ percentile:  M > 7.69  W > 8.88 | **Without NAFLD:**  ASMI, n=140 (3.4%)  HGS, n=280 (6.8%)  no stair climbing, n=322 (7.8%)  **With NAFLD:**  ASMI, n=19 (1.6%)  HGS, n=79 (6.6%)  no stair climbing, n=119 (9.9%) | **Without NAFLD:**  n=387 (9.4%)  **With NAFLD:**  n=169 (14.0%) | Low muscle volume coupled with high muscle fat is a highly vulnerable phenotype within NAFLD, associated with high prevalence of metabolic comorbidity and poor function (arthritis, pain, low overall fitness, and neurological disorders) |

**Legend:** NASH, non-alcoholic steatohepatitis; NAFLD, non-alcoholic fatty liver disease; M, men; W, women; SO, sarcopenic obesity; DXA, dual-energy X-ray absorptiometry; BIA, bioimpedance analysis; US, ultrasound; CT, computed tomography; MRI, magnetic resonance imaging; BMI, body mass index; WC, waist circumference; SMM, skeletal muscle mass; SMI, skeletal muscle index; ASM, appendicular skeletal mass (sum of muscle mass in Kg of both arms and legs); ASMI, appendicular skeletal mass adjusted to height²; ASM-to-BMI, appendicular skeletal mass adjusted to BMI; ASM-to-weight, appendicular skeletal mass adjusted to body weight; HGS, handgrip strength; HGS-to-BMI, handgrip strength adjusted to BMI; SMA-L3, sectional muscle area at the level of the 3^rd^ lumbar vertebra; SMI-L3, skeletal muscle index (SMA adjusted by height²); SMA at L3-to-BMI, sectional muscle area adjusted to BMI; MFI, muscle-fat infiltration; FFM , fat free mass; FM, fat mass; FMI, fat mass index (fat mass adjusted to height²); FFMV, fat-tissue free muscle volume; AGR, android-to-gynoid fat ratio; VFA, visceral fat area; VAT, visceral adipose tissue; SATI, subcutaneous adipose tissue index; VATI, visceral adipose tissue index; SMI + SATI + VATI adjusted to heigth² = body composition area (cm²/m²); RMM, relative muscle mass (sum of muscle mass with fat mass, both in Kg); Δ ASM or Δ FM, changes in body composition, respectively in appendicular skeletal mass, and fat mass; HOMA-IR, homeostasis model assessment of insulin resistance; F2-F3, grade 2 or 3 of liver fibrosis; FibroScan®, transient hepatic elastography; LAI, liver attenuation index; CAP, controlled attenuation parameter; LSM, liver stiffness measurements; AST, aspartate transaminase; ALT, alanine transaminase; AST:ALT ratio, aspartate adjusted to alanine transaminase; NFS, NAFLD fibrosis score; LFS, NAFLD liver fat score; CNS, comprehensive NAFLD score; FIB-4, fibrosis-4-index; HSI, hepatic steatosis index; FLI, Fatty liver index; L:S ratio, liver-spleen attenuation ratio; PDFF, liver proton density fat fraction; NA, not applicable.

Bibliography

1. Yu R, Shi Q, Liu L, Chen L. Relationship of sarcopenia with steatohepatitis and advanced liver fibrosis in non-alcoholic fatty liver disease: a metaanalysis. BMC Gastroenterol. (2018) 18:51. doi: 10.1186/s12876-018-0776-0
2. Wijarnpreecha K, Panjawatanan P, Thongprayoon C, Jaruvongvanich V, Ungprasert P. Sarcopenia and risk of nonalcoholic fatty liver disease: a meta-analysis. Saudi J Gastroenterol Off J Saudi Gastroenterol Assoc. (2018) 24:12–7. doi: 10.4103/sjg.SJG_237_17
3. Pan X, Han Y, Zou T, Zhu G, Xu K, Zheng J, et al. Sarcopenia contributes to the progression of nonalcoholic fatty liver disease-related fibrosis: a metaanalysis. Dig Dis Basel Switz. (2018) 36:427–36. doi: 10.1159/000491015
4. Cai C, Song X, Chen Y, Chen X, Yu C. Relationship between relative skeletal muscle mass and nonalcoholic fatty liver disease: a systematic review and meta-analysis. Hepatol Int. (2020) 14:115–26. doi: 10.1007/s12072-019-09964-1
5. Moon JS, Yoon JS, Won KC, Lee HW. The role of skeletal muscle in development of nonalcoholic Fatty liver disease. Diabetes Metab J. (2013) 37:278–85. doi: 10.4093/dmj.2013.37.4.278
6. Hong HC, Hwang SY, Choi HY, Yoo HJ, Seo JA, Kim SG, et al. Relationship between sarcopenia and nonalcoholic fatty liver disease: the Korean Sarcopenic Obesity Study. Hepatol Baltim Md. (2014) 59:1772–8. doi: 10.1002/hep.26716
7. Hashimoto Y, Osaka T, Fukuda T, Tanaka M, Yamazaki M, Fukui M. The relationship between hepatic steatosis and skeletal muscle mass index in men with type 2 diabetes. *Endocr J*  (2016) 63:877–884. doi:10.1507/endocrj.EJ16-0124
8. Lee Y, Kim SU, Song K, Park JY, Kim DY, Ahn SH, et al. Sarcopenia is associated with significant liver fibrosis independently of obesity and insulin resistance in nonalcoholic fatty liver disease: nationwide surveys (KNHANES 2008–2011). Hepatol Baltim Md. (2016) 63:776–86. doi: 10.1002/hep.2 8376
9. Kim HY, Kim CW, Park C-H, Choi JY, Han K, Merchant AT, Park Y-M. Low skeletal muscle mass is associated with non-alcoholic fatty liver disease in Korean adults: the Fifth Korea National Health and Nutrition Examination Survey. *Hepatobiliary Pancreat Dis Int HBPD INT* (2016) 15:39–47. doi:10.1016/s1499-3872(15)60030-3
10. Koo BK, Kim D, Joo SK, Kim JH, Chang MS, Kim BG, et al. Sarcopenia is an independent risk factor for non-alcoholic steatohepatitis and significant fibrosis. J Hepatol. (2017) 66:123–31. doi: 10.1016/j.jhep.2016.08.019
11. Kim G, Lee S-E, Lee Y-B, Jun JE, Ahn J, Bae JC, et al. Relationship between relative skeletal muscle mass and nonalcoholic fatty liver disease: a 7-year longitudinal study. Hepatol Baltim Md. (2018) 68:1755–68. doi: 10.1002/hep.30049
12. Zhai Y, Xiao Q, Miao J. The Relationship between NAFLD and Sarcopenia in Elderly Patients. *Can J Gastroenterol Hepatol* (2018) 2018:5016091. doi:10.1155/2018/5016091
13. Wijarnpreecha K, Kim D, Raymond P, Scribani M, Ahmed A. Associations between sarcopenia and nonalcoholic fatty liver disease and advanced fibrosis in the USA. *Eur J Gastroenterol Hepatol* (2019) 31:1121–1128. doi:10.1097/MEG.0000000000001397
14. Peng T-C, Wu L-W, Chen W-L, Liaw F-Y, Chang Y-W, Kao T-W. Nonalcoholic fatty liver disease and sarcopenia in a Western population (NHANES III): The importance of sarcopenia definition. *Clin Nutr Edinb Scotl* (2019) 38:422–428. doi:10.1016/j.clnu.2017.11.021
15. Golabi P, Gerber L, Paik JM, Deshpande R, de Avila L, Younossi ZM. Contribution of sarcopenia and physical inactivity to mortality in people

with non-alcoholic fatty liver disease. JHEP Rep Innov Hepatol. (2020)2:100171. doi: 10.1016/j.jhepr.2020.100171

1. Tandon P, Ney M, Irwin I, Ma MM, Gramlich L, Bain VG, Esfandiari N, Baracos V, Montano-Loza AJ, Myers RP. Severe muscle depletion in patients on the liver transplant wait list: its prevalence and independent prognostic value. *Liver Transplant Off Publ Am Assoc Study Liver Dis Int Liver Transplant Soc* (2012) 18:1209–1216. doi:10.1002/lt.23495
2. Cruz RJ, Dew MA, Myaskovsky L, Goodpaster B, Fox K, Fontes P, DiMartini A. Objective radiologic assessment of body composition in patients with end-stage liver disease: going beyond the BMI. *Transplantation* (2013) 95:617–622. doi:10.1097/TP.0b013e31827a0f27
3. Issa D, Alkhouri N, Tsien C, Shah S, Lopez R, McCullough A, et al. Presence of sarcopenia (muscle wasting) in patients with nonalcoholic steatohepatitis. Hepatol Baltim Md. (2014) 60:428–9. doi: 10.1002/hep.26908
4. Montano-Loza AJ, Meza-Junco J, Baracos VE, Prado CMM, Ma M, Meeberg G, et al. Severe muscle depletion predicts postoperative length of stay but is not associated with survival after liver transplantation. Liver Transplant Off Publ Am Assoc Study Liver Dis Int Liver Transplant Soc. (2014) 20:640–8. doi: 10.1002/lt.23863
5. Carias S, Castellanos AL, Vilchez V, Nair R, Dela Cruz AC, Watkins J, et al. Nonalcoholic steatohepatitis is strongly associated with sarcopenic obesity in patients with cirrhosis undergoing liver transplant evaluation. J Gastroenterol Hepatol. (2016) 31:628–33. doi: 10.1111/jgh.13166
6. Carey EJ, Lai JC, Wang CW, Dasarathy S, Lobach I, Montano-Loza AJ, Dunn MA, Fitness, Life Enhancement, and Exercise in Liver Transplantation Consortium. A multicenter study to define sarcopenia in patients with end-stage liver disease. *Liver Transplant Off Publ Am Assoc* *Study Liver Dis Int Liver Transplant Soc* (2017) 23:625–633. doi:10.1002/lt.24750
7. Begini P, Gigante E, Antonelli G, Carbonetti F, Iannicelli E, Anania G, Imperatrice B, Pellicelli AM, Fave GD, Marignani M. Sarcopenia predicts reduced survival in patients with hepatocellular carcinoma at first diagnosis. *Ann Hepatol* (2017) 16:107–114. doi:10.5604/16652681.1226821
8. Golse N, Bucur PO, Ciacio O, Pittau G, Sa Cunha A, Adam R, Castaing D, Antonini T, Coilly A, Samuel D, et al. A new definition of sarcopenia in patients with cirrhosis undergoing liver transplantation. *Liver Transplant Off Publ Am Assoc Study Liver Dis Int Liver Transplant* *Soc* (2017) 23:143–154. doi:10.1002/lt.24671
9. Montano-Loza AJ, Mazurak VC, Ebadi M, Meza-Junco J, Sawyer MB, Baracos VE, Kneteman N. Visceral adiposity increases risk for hepatocellular carcinoma in male patients with cirrhosis and recurrence after liver transplant. *Hepatol Baltim Md* (2018) 67:914–923. doi:10.1002/hep.29578
10. Bhanji RA, Moctezuma-Velazquez C, Duarte-Rojo A, Ebadi M, Ghosh S, Rose C, Montano-Loza AJ. Myosteatosis and sarcopenia are associated with hepatic encephalopathy in patients with cirrhosis. *Hepatol Int* (2018) 12:377–386. doi:10.1007/s12072-018-9875-9
11. Vidot H, Kline K, Cheng R, Finegan L, Lin A, Kempler E, Strasser SI, Bowen DG, McCaughan GW, Carey S, et al. The Relationship of Obesity, Nutritional Status and Muscle Wasting in Patients Assessed for Liver Transplantation. *Nutrients* (2019) 11:E2097. doi:10.3390/nu11092097
12. Dasarathy J, Periyalwar P, Allampati S, Bhinder V, Hawkins C, Brandt P, Khiyami A, McCullough AJ, Dasarathy S. Hypovitaminosis D is associated with increased whole body fat mass and greater severity of non-alcoholic fatty liver disease. *Liver Int Off J Int Assoc Study* *Liver* (2014) 34:e118-127. doi:10.1111/liv.12312
13. Lee Y-H, Jung KS, Kim SU, Yoon H-J, Yun YJ, Lee B-W, et al. Sarcopaenia is associated with NAFLD independently of obesity and insulin resistance: nationwide surveys (KNHANES 2008–2011). J Hepatol. (2015) 63:486–93. doi: 10.1016/j.jhep.2015.02.051
14. Shen H, Liangpunsakul S. Association between sarcopenia and prevalence of nonalcoholic fatty liver disease: a cross-sectional study from the Third National Health and Nutrition Examination Survey (Mo1555*). Gastroenterology* (2016) 150: S1143–S4. doi: 10.1016/s0016-5085(16)33859-8
15. Petta S, Ciminnisi S, Di Marco V, Cabibi D, Cammà C, Licata A, et al. Sarcopenia is associated with severe liver fibrosis in patients with nonalcoholic fatty liver disease. Aliment Pharmacol Ther. (2017) 45:510–8. doi: 10.1111/apt.13889
16. Rachakonda V, Wills R, DeLany JP, Kershaw EE, Behari J. Differential impact of weight loss on nonalcoholic fatty liver resolution in a North American Cohort with obesity. Obes Silver Spring Md. (2017) 25:1360–8. doi: 10.1002/oby.21890
17. Choe EK, Kang HY, Park B, Yang JI, Kim JS. The Association between Nonalcoholic Fatty Liver Disease and CT-Measured Skeletal Muscle Mass. *J Clin Med* (2018) 7: E310. doi:10.3390/jcm7100310
18. Lee MJ, Kim E-H, Bae S-J, Kim G-A, Park SW, Choe J, et al. Age-related decrease in skeletal muscle mass is an independent risk factor for incident nonalcoholic fatty liver disease: a 10-year retrospective cohort study. Gut Liver. (2019) 13:67–76. doi: 10.5009/gnl18070
19. Alferink LJM, Trajanoska K, Erler NS, Schoufour JD, de Knegt RJ, Ikram MA, Janssen HLA, Franco OH, Metselaar HJ, Rivadeneira F, et al. Nonalcoholic Fatty Liver Disease in The Rotterdam Study: About Muscle Mass, Sarcopenia, Fat Mass, and Fat Distribution. *J Bone Miner* *Res Off J Am Soc Bone Miner Res* (2019) 34:1254–1263. doi:10.1002/jbmr.
20. Kang S, Moon MK, Kim W, Koo BK. Association between muscle strength and advanced fibrosis in non-alcoholic fatty liver disease: a Korean nationwide survey. *J Cachexia Sarcopenia Muscle* (2020) 11:1232–1241. doi:10.1002/jcsm.12598
21. Gan D, Wang L, Jia M, Ru Y, Ma Y, Zheng W, Zhao X, Yang F, Wang T, Mu Y, et al. Low muscle mass and low muscle strength associate with nonalcoholic fatty liver disease. *Clin Nutr*  *Edinb Scotl* (2020) 39:1124–1130. doi:10.1016/j.clnu.2019.04.023
22. De Munck TJI, Verhaegh P, Lodewick T, Bakers F, Jonkers D, Masclee AAM, et al. Myosteatosis in nonalcoholic fatty liver disease: an exploratory study. Clin Res Hepatol Gastroenterol. (2021) 45:101500. doi: 10.1016/j.clinre.2020.06.021
23. Pacifico L, Perla FM, Andreoli G, Grieco R, Pierimarchi P, Chiesa C. Nonalcoholic Fatty Liver Disease Is Associated With Low Skeletal Muscle Mass in Overweight/Obese Youths. *Front Pediatr* (2020) 8:158. doi:10.3389/fped.2020.00158
24. Wang Y-M, Zhu K-F, Zhou W-J, Zhang Q, Deng D-F, Yang Y-C, Lu W-W, Xu J, Yang Y-M. Sarcopenia is associated with the presence of nonalcoholic fatty liver disease in Zhejiang Province, China: a cross-sectional observational study. *BMC Geriatr* (2021) 21:55.
25. Wijarnpreecha K, Aby ES, Ahmed A, KimD. Association between sarcopenic obesity and nonalcoholic fatty liver disease and fibrosis detected by fibroscan. J Gastrointest Liver Dis JGLD. (2021) 30:227–32. doi: 10.15403/jgld-3323
26. Kang M-K, Baek J-H, Kweon Y-O, Tak W-Y, Jang S-Y, Lee Y-R, Hur K, Kim G, Lee H-W, Han M-H, et al. Association of Skeletal Muscle and Adipose Tissue Distribution with Histologic Severity of Non-Alcoholic Fatty Liver. *Diagn Basel Switz* (2021) 11:1061. doi:10.3390/diagnostics11061061
27. Nachit M, Kwanten WJ, Thissen J-P, Op De Beeck B, Van Gaal L, Vonghia L, et al. Muscle fat content is strongly associated with NASH: a longitudinal study in patients with morbid obesity. J Hepatol. (2021) 75:292–301. doi: 10.1016/j.jhep.2021.02.037
28. Linge J, Ekstedt M, Dahlqvist Leinhard O. Adverse muscle composition is linked to poor functional performance and metabolic comorbidities in NAFLD. JHEP Rep Innov Hepatol. (2021)3:100197. doi: 10.1016/j.jhepr.2020.100197
