# Supplementary material for: Impact of Sarcopenia on the Severity of the Liver Damage in Patients With Non-alcoholic Fatty Liver Disease
Source: Front Nutr. 2022 Jan 17;8:774030. doi: 10.3389/fnut.2021.774030 (PMC8802760; doi:10.3389/fnut.2021.774030)
Supplement: Supplementary file 2 [file Data_Sheet_2.docx]

Bibliography Supplementary material (Table 1,2,3)

1. Yu R, Shi Q, Liu L, Chen L. Relationship of sarcopenia with steatohepatitis and advanced liver fibrosis in non-alcoholic fatty liver disease: a metaanalysis. BMC Gastroenterol. (2018) 18:51. doi: 10.1186/s12876-018-0776-0
2. Wijarnpreecha K, Panjawatanan P, Thongprayoon C, Jaruvongvanich V, Ungprasert P. Sarcopenia and risk of nonalcoholic fatty liver disease: a meta-analysis. Saudi J Gastroenterol Off J Saudi Gastroenterol Assoc. (2018) 24:12–7. doi: 10.4103/sjg.SJG_237_17
3. Pan X, Han Y, Zou T, Zhu G, Xu K, Zheng J, et al. Sarcopenia contributes to the progression of nonalcoholic fatty liver disease-related fibrosis: a metaanalysis. Dig Dis Basel Switz. (2018) 36:427–36. doi: 10.1159/000491015
4. Cai C, Song X, Chen Y, Chen X, Yu C. Relationship between relative skeletal muscle mass and nonalcoholic fatty liver disease: a systematic review and meta-analysis. Hepatol Int. (2020) 14:115–26. doi: 10.1007/s12072-019-09964-1
5. Moon JS, Yoon JS, Won KC, Lee HW. The role of skeletal muscle in development of nonalcoholic Fatty liver disease. Diabetes Metab J. (2013) 37:278–85. doi: 10.4093/dmj.2013.37.4.278
6. Hong HC, Hwang SY, Choi HY, Yoo HJ, Seo JA, Kim SG, et al. Relationship between sarcopenia and nonalcoholic fatty liver disease: the Korean Sarcopenic Obesity Study. Hepatol Baltim Md. (2014) 59:1772–8. doi: 10.1002/hep.26716
7. Hashimoto Y, Osaka T, Fukuda T, Tanaka M, Yamazaki M, Fukui M. The relationship between hepatic steatosis and skeletal muscle mass index in men with type 2 diabetes. *Endocr J*  (2016) 63:877–884. doi:10.1507/endocrj.EJ16-0124
8. Lee Y, Kim SU, Song K, Park JY, Kim DY, Ahn SH, et al. Sarcopenia is associated with significant liver fibrosis independently of obesity and insulin resistance in nonalcoholic fatty liver disease: nationwide surveys (KNHANES 2008–2011). Hepatol Baltim Md. (2016) 63:776–86. doi: 10.1002/hep.2 8376
9. Kim HY, Kim CW, Park C-H, Choi JY, Han K, Merchant AT, Park Y-M. Low skeletal muscle mass is associated with non-alcoholic fatty liver disease in Korean adults: the Fifth Korea National Health and Nutrition Examination Survey. *Hepatobiliary Pancreat Dis Int HBPD INT* (2016) 15:39–47. doi:10.1016/s1499-3872(15)60030-3
10. Koo BK, Kim D, Joo SK, Kim JH, Chang MS, Kim BG, et al. Sarcopenia is an independent risk factor for non-alcoholic steatohepatitis and significant fibrosis. J Hepatol. (2017) 66:123–31. doi: 10.1016/j.jhep.2016.08.019
11. Kim G, Lee S-E, Lee Y-B, Jun JE, Ahn J, Bae JC, et al. Relationship between relative skeletal muscle mass and nonalcoholic fatty liver disease: a 7-year longitudinal study. Hepatol Baltim Md. (2018) 68:1755–68. doi: 10.1002/hep.30049
12. Zhai Y, Xiao Q, Miao J. The Relationship between NAFLD and Sarcopenia in Elderly Patients. *Can J Gastroenterol Hepatol* (2018) 2018:5016091. doi:10.1155/2018/5016091
13. Wijarnpreecha K, Kim D, Raymond P, Scribani M, Ahmed A. Associations between sarcopenia and nonalcoholic fatty liver disease and advanced fibrosis in the USA. *Eur J Gastroenterol Hepatol* (2019) 31:1121–1128. doi:10.1097/MEG.0000000000001397
14. Peng T-C, Wu L-W, Chen W-L, Liaw F-Y, Chang Y-W, Kao T-W. Nonalcoholic fatty liver disease and sarcopenia in a Western population (NHANES III): The importance of sarcopenia definition. *Clin Nutr Edinb Scotl* (2019) 38:422–428. doi:10.1016/j.clnu.2017.11.021
15. Golabi P, Gerber L, Paik JM, Deshpande R, de Avila L, Younossi ZM. Contribution of sarcopenia and physical inactivity to mortality in people

with non-alcoholic fatty liver disease. JHEP Rep Innov Hepatol. (2020)2:100171. doi: 10.1016/j.jhepr.2020.100171

1. Tandon P, Ney M, Irwin I, Ma MM, Gramlich L, Bain VG, Esfandiari N, Baracos V, Montano-Loza AJ, Myers RP. Severe muscle depletion in patients on the liver transplant wait list: its prevalence and independent prognostic value. *Liver Transplant Off Publ Am Assoc Study Liver Dis Int Liver Transplant Soc* (2012) 18:1209–1216. doi:10.1002/lt.23495
2. Cruz RJ, Dew MA, Myaskovsky L, Goodpaster B, Fox K, Fontes P, DiMartini A. Objective radiologic assessment of body composition in patients with end-stage liver disease: going beyond the BMI. *Transplantation* (2013) 95:617–622. doi:10.1097/TP.0b013e31827a0f27
3. Issa D, Alkhouri N, Tsien C, Shah S, Lopez R, McCullough A, et al. Presence of sarcopenia (muscle wasting) in patients with nonalcoholic steatohepatitis. Hepatol Baltim Md. (2014) 60:428–9. doi: 10.1002/hep.26908
4. Montano-Loza AJ, Meza-Junco J, Baracos VE, Prado CMM, Ma M, Meeberg G, et al. Severe muscle depletion predicts postoperative length of stay but is not associated with survival after liver transplantation. Liver Transplant Off Publ Am Assoc Study Liver Dis Int Liver Transplant Soc. (2014) 20:640–8. doi: 10.1002/lt.23863
5. Carias S, Castellanos AL, Vilchez V, Nair R, Dela Cruz AC, Watkins J, et al. Nonalcoholic steatohepatitis is strongly associated with sarcopenic obesity in patients with cirrhosis undergoing liver transplant evaluation. J Gastroenterol Hepatol. (2016) 31:628–33. doi: 10.1111/jgh.13166
6. Carey EJ, Lai JC, Wang CW, Dasarathy S, Lobach I, Montano-Loza AJ, Dunn MA, Fitness, Life Enhancement, and Exercise in Liver Transplantation Consortium. A multicenter study to define sarcopenia in patients with end-stage liver disease. *Liver Transplant Off Publ Am Assoc* *Study Liver Dis Int Liver Transplant Soc* (2017) 23:625–633. doi:10.1002/lt.24750
7. Begini P, Gigante E, Antonelli G, Carbonetti F, Iannicelli E, Anania G, Imperatrice B, Pellicelli AM, Fave GD, Marignani M. Sarcopenia predicts reduced survival in patients with hepatocellular carcinoma at first diagnosis. *Ann Hepatol* (2017) 16:107–114. doi:10.5604/16652681.1226821
8. Golse N, Bucur PO, Ciacio O, Pittau G, Sa Cunha A, Adam R, Castaing D, Antonini T, Coilly A, Samuel D, et al. A new definition of sarcopenia in patients with cirrhosis undergoing liver transplantation. *Liver Transplant Off Publ Am Assoc Study Liver Dis Int Liver Transplant* *Soc* (2017) 23:143–154. doi:10.1002/lt.24671
9. Montano-Loza AJ, Mazurak VC, Ebadi M, Meza-Junco J, Sawyer MB, Baracos VE, Kneteman N. Visceral adiposity increases risk for hepatocellular carcinoma in male patients with cirrhosis and recurrence after liver transplant. *Hepatol Baltim Md* (2018) 67:914–923. doi:10.1002/hep.29578
10. Bhanji RA, Moctezuma-Velazquez C, Duarte-Rojo A, Ebadi M, Ghosh S, Rose C, Montano-Loza AJ. Myosteatosis and sarcopenia are associated with hepatic encephalopathy in patients with cirrhosis. *Hepatol Int* (2018) 12:377–386. doi:10.1007/s12072-018-9875-9
11. Vidot H, Kline K, Cheng R, Finegan L, Lin A, Kempler E, Strasser SI, Bowen DG, McCaughan GW, Carey S, et al. The Relationship of Obesity, Nutritional Status and Muscle Wasting in Patients Assessed for Liver Transplantation. *Nutrients* (2019) 11:E2097. doi:10.3390/nu11092097
12. Dasarathy J, Periyalwar P, Allampati S, Bhinder V, Hawkins C, Brandt P, Khiyami A, McCullough AJ, Dasarathy S. Hypovitaminosis D is associated with increased whole body fat mass and greater severity of non-alcoholic fatty liver disease. *Liver Int Off J Int Assoc Study* *Liver* (2014) 34:e118-127. doi:10.1111/liv.12312
13. Lee Y-H, Jung KS, Kim SU, Yoon H-J, Yun YJ, Lee B-W, et al. Sarcopaenia is associated with NAFLD independently of obesity and insulin resistance: nationwide surveys (KNHANES 2008–2011). J Hepatol. (2015) 63:486–93. doi: 10.1016/j.jhep.2015.02.051
14. Shen H, Liangpunsakul S. Association between sarcopenia and prevalence of nonalcoholic fatty liver disease: a cross-sectional study from the Third National Health and Nutrition Examination Survey (Mo1555*). Gastroenterology* (2016) 150: S1143–S4. doi: 10.1016/s0016-5085(16)33859-8
15. Petta S, Ciminnisi S, Di Marco V, Cabibi D, Cammà C, Licata A, et al. Sarcopenia is associated with severe liver fibrosis in patients with nonalcoholic fatty liver disease. Aliment Pharmacol Ther. (2017) 45:510–8. doi: 10.1111/apt.13889
16. Rachakonda V, Wills R, DeLany JP, Kershaw EE, Behari J. Differential impact of weight loss on nonalcoholic fatty liver resolution in a North American Cohort with obesity. Obes Silver Spring Md. (2017) 25:1360–8. doi: 10.1002/oby.21890
17. Choe EK, Kang HY, Park B, Yang JI, Kim JS. The Association between Nonalcoholic Fatty Liver Disease and CT-Measured Skeletal Muscle Mass. *J Clin Med* (2018) 7: E310. doi:10.3390/jcm7100310
18. Lee MJ, Kim E-H, Bae S-J, Kim G-A, Park SW, Choe J, et al. Age-related decrease in skeletal muscle mass is an independent risk factor for incident nonalcoholic fatty liver disease: a 10-year retrospective cohort study. Gut Liver. (2019) 13:67–76. doi: 10.5009/gnl18070
19. Alferink LJM, Trajanoska K, Erler NS, Schoufour JD, de Knegt RJ, Ikram MA, Janssen HLA, Franco OH, Metselaar HJ, Rivadeneira F, et al. Nonalcoholic Fatty Liver Disease in The Rotterdam Study: About Muscle Mass, Sarcopenia, Fat Mass, and Fat Distribution. *J Bone Miner* *Res Off J Am Soc Bone Miner Res* (2019) 34:1254–1263. doi:10.1002/jbmr.
20. Kang S, Moon MK, Kim W, Koo BK. Association between muscle strength and advanced fibrosis in non-alcoholic fatty liver disease: a Korean nationwide survey. *J Cachexia Sarcopenia Muscle* (2020) 11:1232–1241. doi:10.1002/jcsm.12598
21. Gan D, Wang L, Jia M, Ru Y, Ma Y, Zheng W, Zhao X, Yang F, Wang T, Mu Y, et al. Low muscle mass and low muscle strength associate with nonalcoholic fatty liver disease. *Clin Nutr*  *Edinb Scotl* (2020) 39:1124–1130. doi:10.1016/j.clnu.2019.04.023
22. De Munck TJI, Verhaegh P, Lodewick T, Bakers F, Jonkers D, Masclee AAM, et al. Myosteatosis in nonalcoholic fatty liver disease: an exploratory study. Clin Res Hepatol Gastroenterol. (2021) 45:101500. doi: 10.1016/j.clinre.2020.06.021
23. Pacifico L, Perla FM, Andreoli G, Grieco R, Pierimarchi P, Chiesa C. Nonalcoholic Fatty Liver Disease Is Associated With Low Skeletal Muscle Mass in Overweight/Obese Youths. *Front Pediatr* (2020) 8:158. doi:10.3389/fped.2020.00158
24. Wang Y-M, Zhu K-F, Zhou W-J, Zhang Q, Deng D-F, Yang Y-C, Lu W-W, Xu J, Yang Y-M. Sarcopenia is associated with the presence of nonalcoholic fatty liver disease in Zhejiang Province, China: a cross-sectional observational study. *BMC Geriatr* (2021) 21:55.
25. Wijarnpreecha K, Aby ES, Ahmed A, KimD. Association between sarcopenic obesity and nonalcoholic fatty liver disease and fibrosis detected by fibroscan. J Gastrointest Liver Dis JGLD. (2021) 30:227–32. doi: 10.15403/jgld-3323
26. Kang M-K, Baek J-H, Kweon Y-O, Tak W-Y, Jang S-Y, Lee Y-R, Hur K, Kim G, Lee H-W, Han M-H, et al. Association of Skeletal Muscle and Adipose Tissue Distribution with Histologic Severity of Non-Alcoholic Fatty Liver. *Diagn Basel Switz* (2021) 11:1061. doi:10.3390/diagnostics11061061
27. Nachit M, Kwanten WJ, Thissen J-P, Op De Beeck B, Van Gaal L, Vonghia L, et al. Muscle fat content is strongly associated with NASH: a longitudinal study in patients with morbid obesity. J Hepatol. (2021) 75:292–301. doi: 10.1016/j.jhep.2021.02.037
28. Linge J, Ekstedt M, Dahlqvist Leinhard O. Adverse muscle composition is linked to poor functional performance and metabolic comorbidities in NAFLD. JHEP Rep Innov Hepatol. (2021)3:100197. doi: 10.1016/j.jhepr.2020.100197
